# Functional morphology and biomechanics of the locomotor apparatus in the large Late Triassic carnivore *Postosuchus kirkpatricki* (Archosauria: Rauisuchidae)

**DOI:** 10.1111/joa.70189

**Published:** 2026-06-05

**Authors:** John R. Hutchinson, Emily Faughey, Matthew Humpage, Tristan Dupuis, Oliver E. Demuth, Romain Pintore, Francois Clarac

**Affiliations:** ^1^ Structure & Motion Laboratory, Department of Comparative Biomedical Sciences Royal Veterinary College Hatfield UK; ^2^ Northern Rogue Studios Inverness UK; ^3^ Institute of Molecular Plant Sciences, School of Biological Sciences University of Edinburgh, Max Born Crescent Edinburgh UK; ^4^ Ecole Nationale Veterinaire Toulouse Toulouse France; ^5^ Clare College University of Cambridge Cambridge UK; ^6^ Department of Earth Sciences University of Cambridge Cambridge UK; ^7^ Mécanismes adaptatifs et évolution (UMR 7179 ‐ MECADEV), Muséum National d’Histoire Naturelle, Paris, CNRS, CP 55 Paris France; ^8^ Institut des Sciences de la Terre de Paris Sorbonne Université, CNRS, (UMR 7193 ‐ ISTEP), Campus Pierre et Marie Curie Paris France; ^9^ Institut de Physique du Globe de Paris Université Paris Cité, CNRS, (UMR 7154 ‐ IPGP) Paris France; ^10^ Centre de Recherche en Paléontologie – Paris (CR2P), Muséum national d’Histoire naturelle Sorbonne Université, CNRS, CP 38 Paris France

**Keywords:** bipedalism, functional morphology, locomotion, musculoskeletal model, Pseudosuchia, Triassic

## Abstract

*Postosuchus kirkpatricki* was a large pseudosuchian archosaur from the Late Triassic period in North America. It is among several pseudosuchians proposed to have had derived aspects of locomotor function such as bipedalism or digitigrady, rather than plesiomorphic quadrupedalism or plantigrady, but disputes and inconsistencies about these propositions remain. These lingering disputes need resolution in order to formulate broader inferences about the evolution of bipedalism, limb posture, athleticism, and the end‐Triassic mass extinctions. Here, we use 3D musculoskeletal modelling to address the disputes via a deep critical review of available evidence via multiple methods. We conclude that it is uncertain if *Postosuchus* spp. was quadrupedal or bipedal, plantigrade or digitigrade, due to conflicting evidence. Our analyses also reconstruct pelvic limb musculature that was relatively three times as massive as that in a similar‐sized Nile crocodile, whereas the caudofemoralis was smaller than expected due to the gracile tail of *Postosuchus*. Aspects of hindlimb myology and morphofunctional analyses of the hindlimb joints suggest a mix of traits that are plesiomorphic archosaurian, derived “rauisuchian” and singular for *Postosuchus*. Our extensive modelling procedure and synthesis of current evidence forms a foundation for future studies such as predictive simulations or ichnological evidence of locomotor function.

## Graphical abstract

A three‐dimensional model is used to analyze the locomotor biomechanics of the large Late Triassic archosaurian reptile *Postosuchus kirkpatricki*. The study finds that it is more uncertain than previously concluded whether it was quadrupedal or bipedal, and plantigrade or digitigrade, but it clearly had locomotor specializations including large hindlimb muscles.

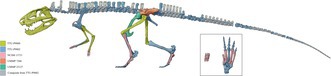

## 1 INTRODUCTION

*Postosuchus* was one of the largest fully terrestrial archosaurs of the Late Triassic period. There are two species known to date, both from North America: *P. kirkpatricki* from the western continent, mainly from Texas (Dockum Group; mid‐Norian age, ~215 Mya) but also New Mexico and Arizona; and *P. alisonae* from North Carolina (Durham sub‐basin; late Carnian to early Norian age). *P. kirkpatricki* is known mainly from two partial, associated skeletons (Texas Tech University Paleontology Division collection specimen numbers TTU‐P 9000 and 9002; Chatterjee, 1985), with a complex history of reinterpretations of those and other putative specimens of the taxon by subsequent studies. TTU‐P 9000 is an adult or subadult, whereas TTU‐P 9002 is a smaller, presumably juvenile specimen (e.g., Chatterjee, 1985). *P. alisonae* is known from a partial, near‐adult skeleton (North Carolina Museum of Natural Sciences specimen number NCSM 13731l; formerly UNC 15575; Peyer et al., 2008). Although Chatterjee (1985) named and described the genus, Case (1922, 1932, 1943) had reported on the first specimens already, noting a partial pelvis with a “radically different” pubis characterized by its narrowness and “hook‐like” distal expansion (Case, 1943; University of Michigan Museum of Paleontology specimen number UMMP VP 23127); and assigning a braincase to the theropod dinosaur *Coelophysis* (Case, 1922; UMMP VP 7473). Chatterjee (1985) referred all of these remains and an ilium (Texas Memorial Museum specimen number TMM 31025‐12) to the new genus, placed within the “rauisuchian” family Poposauridae, although subsequent studies showed potential synapomorphies uniting poposaur(o)ids and *Postosuchus* to be convergent evolution or due to incorrect referral of specimens (e.g., Benton & Clark, 1988; Brusatte et al., 2010; Gauthier, 1986; Juul, 1994; Nesbitt, 2011). Chatterjee's (1985) argument that *Postosuchus* was close to the ancestry of tyrannosaurs has long since been dismissed, but is a prime example of homoplasy of that body plan (i.e., large head; small forelimbs) among bipedal carnivorous archosaurs (e.g., Bates & Schachner, 2012; Nesbitt & Norell, 2006). There were descriptions or reassignments (e.g., Long & Murry, 1995; Nesbitt, 2011; Novak, 2004; Weinbaum, 2002) of material from the aforementioned and other *Postosuchus* specimens (e.g., numerous specimens from the University of California Museum of Paleontology [Berkeley, CA, USA]) until Weinbaum's (2011, 2013) detailed descriptions, along with Peyer et al.'s (2008) description of *P. alisonae*, followed by Marsh et al.'s (2026) recent description of new material. Here, we use “*Postosuchus*” throughout but focus on *P. kirkpatricki*, and where *P. alisonae* is referred to, we make that explicit.

**FIGURE 1:**
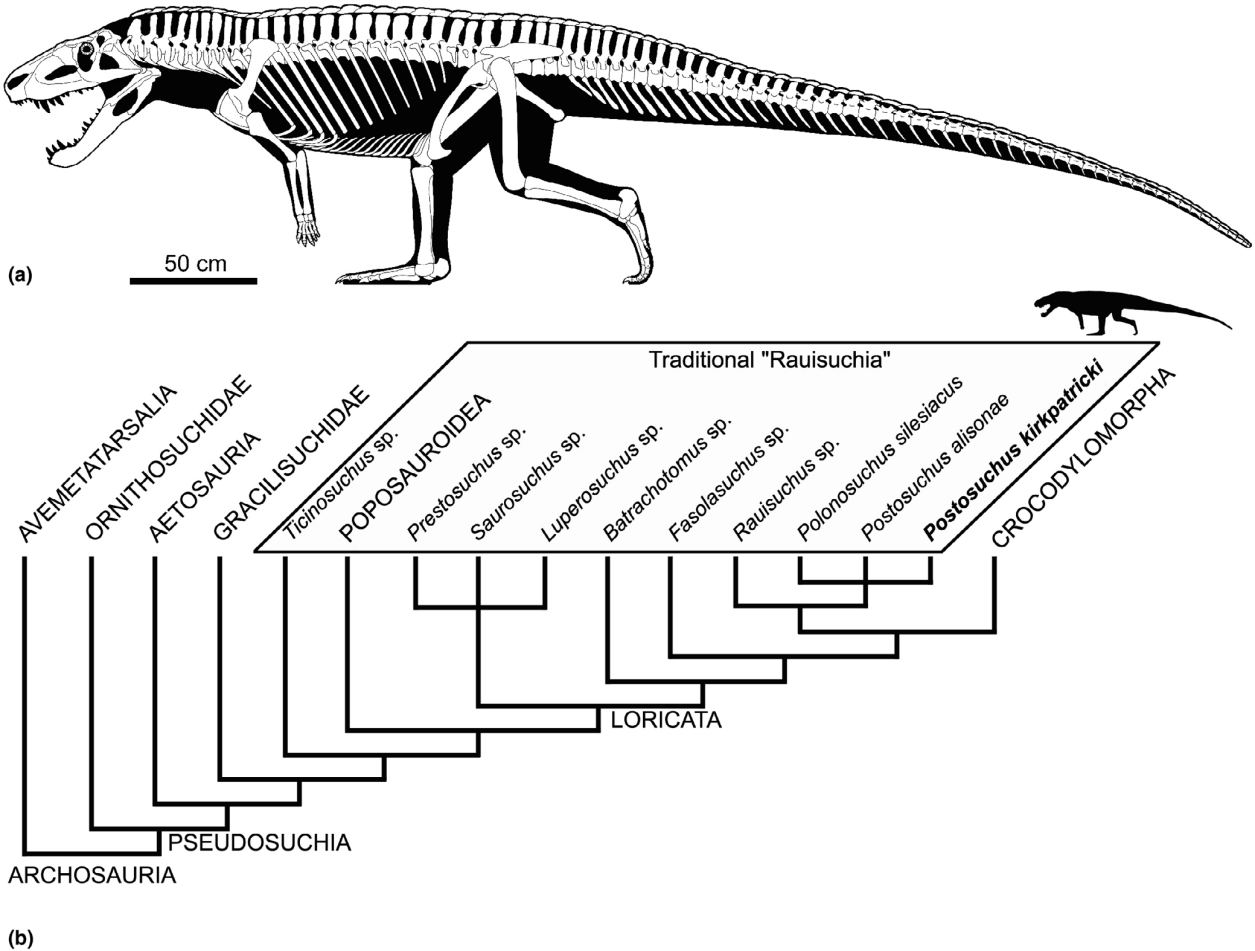
(a) Skeletal reconstruction of *Postosuchus kirkpatricki* in left lateral view, by Scott Hartman, used with permission; reconstructed in a hypothetical plantigrade foot pose and bipedalism. (b) Composite, simplified phylogeny of Archosauria (following Ezcurra et al., 2017, 2020; Müller, 2024; Nesbitt, 2011) showing the most commonly suggested phylogenetic position of *Postosuchus* spp. recently (see text for details).

The Triassic Period was a time of flourishing of locomotor (and other functional and behavioral) specializations in the clade Archosauria, with numerous independent “experiments” in major traits such as bipedalism (e.g., Bates & Schachner, 2012; Charig, 1972; Cuff et al., 2022; Demuth et al., 2023; Gauthier et al., 2011; Grinham et al., 2019; Kubo & Kubo, 2012; Nesbitt & Norell, 2006; Persons & Currie, 2017). *Postosuchus* is important for understanding locomotor diversity in Triassic archosaurs because of its large body size (4–6 m long and >200 kg; Chatterjee, 1985), its possible bipedalism, and its many unusual postcranial features noted above. As *Postosuchus* likely is not closely related to “known” bipedal suchian archosaurs such as certain Poposauroidea or the enigmatic *Smok* (Niedźwiedzki et al., 2011), understanding its locomotor morphology and function is valuable for assessing how greatly bipedal (or biped‐like) suchians varied in their locomotion versus variation in early ornithodiran/dinosaurian archosaurs. *Postosuchus* is also important for reconstructing the polarity of evolution within suchian archosaurs, especially because it is often recovered as closely related to Crocodylomorpha (Figure 1; e.g., Benton & Clark, 1988; Brusatte et al., 2010; Gauthier, 1986; Juul, 1994; Nesbitt, 2011; Nesbitt et al., 2017; Weinbaum, 2011). Thus, whether it is inferred as bipedal versus quadrupedal, or plantigrade versus digitigrade, its stance and gait impact key questions about what the ancestral stance and gait of Crocodylomorpha was; for example, were early crocodylomorphs bipedal or not (reviewed by Cuff et al., 2022 and Spiekman et al., 2024)? Admittedly, some studies have recovered *Postosuchus* (with other “Rauisuchoidea”) as much more distantly related to Crocodylomorpha (e.g., Brusatte et al., 2010; Butler et al., 2011). Even then, *Postosuchus* is relevant for estimating how many times other important attributes such as digitigrade autopodia or “pillar‐erect” hip joint articulations evolved in archosaurs (e.g., Bates & Schachner, 2012; Demuth et al., 2020; Farlow et al., 2014).

Recognition of *Postosuchus* as distinct from most other archosaurs was enabled by diagnostic apomorphies such as a reduced manus with blunt unguals on digits III and IV (Chatterjee, 1985; Peyer et al., 2008; Weinbaum, 2013). Furthermore, some postcranial traits have led to inferences about the functional morphology, behavior and palaeoecology of *Postosuchus*. Long and Murry (1995) concluded that some of the *Postosuchus* postcranial material used in Chatterjee's (1985) reconstruction was from a different taxon, “*Chatterjeea*” (now known as *Shuvosaurus*, a poposaurid [Figure 1]; e.g., Brusatte et al., 2010). Based on the similarities of the limb proportions of actual *Postosuchus* specimens to the presumably quadrupedal “rauisuchian” *Ticinosuchus* (Figure 1), and quadrupedal *Synaptichnium* trackways attributed to an animal like *Ticinosuchus*, Long and Murry (1995) favored quadrupedalism for *Postosuchus*. Peyer et al. (2008) noted the robust glenoid with restricted mobility, inferring that it indicated a more erect forelimb posture. They considered the long humerus and forearm bones to indicate quadrupedal locomotion, albeit perhaps with facultative bipedalism (also Chatterjee, 1985); noting that the short manus was tightly interlocking, which might have enabled a supportive role. In a fairly detailed analysis, Hutson and Hutson (2015) revisited the issue of a rigid manus in *Postosuchus* via comparisons with experimentally manipulated joints of alligators, and concluded that the tongue‐and‐groove articulations of the metacarpals (but not evident in TTU‐P 9002), robust phalanges and flattened articular surfaces in *P. alisonae* are reminiscent of those of quadrupedal dinosaurs such as sauropods, stegosaurs, hadrosaurs, and large ceratopsids (Hutson, 2015; Senter, 2010), hence consistent with a quadrupedal *Postosuchus*. They also described the strong extensor fossae of metacarpophalangeal joints and some phalanges in *Postosuchus*, which allowed digital hyperextension for usage in quadrupedalism, most likely involving a vertically oriented manus (“metacarpogrady”). Conspicuously, like other non‐crocodylomorph archosaurs, *Postosuchus* lacks an elongate radiale and ulnare; derived traits apparently related to specialized quadrupedal function in Crocodylomorpha (e.g., Spiekman, 2023; Spiekman et al., 2024; Walker, 1970). Nesbitt (2011) reviewed the evolution of the orientation of the glenoid in archosauromorphs, commenting that it faces caudoventrally in *Postosuchus* and Crocodylomorpha; this trait likewise is consistent with a more erect forelimb when used in quadrupedalism. Some studies have mentioned or reconstructed *Postosuchus* as being quadrupedal (e.g., Brusatte et al., 2009, 2010; Nesbitt et al., 2013; Parrish, 1986), often without further comment or analysis.

As Weinbaum (2013) noted after reconsidering the problematic nature of the *P. kirkpatricki* specimens, the combined lengths of the humerus and radius/ulna are only little more than the length of the femur, which would favor a bipedal interpretation (e.g., Hartman et al., 2022; but see Kubo & Kubo, 2012, who favored quadrupedalism). The study also commented that the manus was too reduced to likely play a supportive role in quadrupedalism (but Hutson & Hutson, 2015; as above; challenged this point), rejecting Peyer et al.'s (2008) claims that the robust pectoral girdle indicated quadrupedalism. Furthermore, numerous theropods and other bipedal archosaurs (e.g., Nesbitt & Norell, 2006; Schachner et al., 2020) have such robust girdles. More quantitative analytical methods also have addressed the question of bipedalism in *Postosuchus*. Bishop et al. (2020) collated a broad dataset of limb lengths, estimated body masses and estimated center of mass (COM) positions for archosauromorphs and found from this that *Postosuchus* most likely (~85%–100% probability) was bipedal. Contrastingly, Pintore et al. (2022) applied 3D geometric morphometrics methods to the femora of diverse archosauriforms, predicting that specimen TTU‐P 9000 was bipedal but the smaller specimen TTU‐P 9002 was quadrupedal; thus, overall giving ambiguous results for *Postosuchus*. However, their analysis noted a more medially oriented femoral head (37°–62° vs femoral condyles) and a more acute angle between the lateral condyle and the crista tibiofibularis (103°–105°) than quadrupedal archosauriforms tend to have. Somewhat similarly, Hartman et al. (2022) argued that, relative to the bipedal *Poposaurus*, the forelimbs of *Postosuchus* are built in a way more amenable to some weight support such as during standing or slow walking, and that, because the forelimbs transition from relatively longer to shorter across ontogeny in *Postosuchus*, perhaps it shifted its stance from more quadrupedal to more bipedal with adulthood. Weinbaum (2007) studied cranial endocasts (TTU‐P 9002 and UMMP‐7473) and considered these to be evidence for bipedality in *Postosuchus kirkpatricki*, because of the similarity of the caudal region of the brain's morphology to large bipedal theropod dinosaurs, and an enlarged flocculus.

Numerous other traits of the pelvic appendages of *Postosuchus* give seemingly conflicting or ambiguous signals about quadrupedalism versus bipedalism or other broad aspects of hindlimb function. There are two (Weinbaum, 2013), not three or more (e.g., Chatterjee, 1985) sacral vertebrae, whereas most bipedal archosaurs have the latter (Gauthier et al., 2011), for reasons as yet not mechanistically understood in detail. However, the sacral vertebrae have expansive sacral ribs that may have further strengthened the sacral region, providing improved support. The acetabulum is partly open at the ischio‐pubic junction (Juul, 1994; Weinbaum, 2013), which probably is correlated more with changes in acetabular ligaments and an erect posture rather than strictly bipedalism (Tsai & Holliday, 2014). There is a caudally expansive pubic boot that is generally seen in some bipedal early archosaurs (theropods and the poposauroids *Effigia*, *Shuvosaurus*, and *Poposaurus*—and the suchian *Smok*; Niedzwiedzki & Budziszewska‐Karwowska, 2018), perhaps related to support when sitting bipedally. The “rauisuchian” *Batrachotomus* (Figure 1)—which possibly was quadrupedal (Bishop et al., 2020)—however, has a sizable pubic boot (Gower & Schoch, 2009). Some quadrupedal relatives of those bipedal archosaurs with large pubic boots instead have somewhat enlarged pubic knobs (e.g., many “rauisuchians”; the poposauroid *Arizonasaurus*), suggesting a transitional state. However, such pubic boots or knobs are absent in other potentially or “known” bipedal archosaurs such as lagerpetids, *Lagosuchus* and silesaurids, as well as ornithischians, so these structures at best are only correlates of bipedalism.

Finally, the pes (and manus, if quadrupedal) of *Postosuchus* has variably been characterized or depicted as plantigrade (Long & Murry, 1995; Nesbitt et al., 2013; Parrish, 1986; Peyer et al., 2008; Weinbaum, 2013) or digitigrade (Benton & Clark, 1988; Chatterjee, 1985; Juul, 1994; Parrish, 1986). Turner and Gatesy (2023; “complex 2” in their fig. 7) used *Postosuchus* as one example of how archosauriform ankle joints match a morphofunctional continuum from an ancestral 3D rotary form with rotational contributions from many joints toward a derived, more hingelike ankle form with only two main joints (see also Parrish, 1986). Yet essentially all studies accept that the limb posture of *Postosuchus* was strongly erect (adducted) based upon not only glenoid/forelimb morphology but also particularly upon the substantial supra‐acetabular crest on the ilium, and how the ilia are ventrolaterally tilted to more completely cover the femoral head, forming a “pillar‐erect” hip articulation (Benton & Clark, 1988; also Bonaparte, 1984; Parrish, 1986). Bates and Schachner (2012) posited that bipedalism and “pillar‐erect” hips are linked in pseudosuchians (namely *Poposaurus*); analysis of *Postosuchus* can further test that idea, taking into account the morphofunctional differences between different taxa.

Here, we construct a three‐dimensional (3D) biomechanical model of *Postosuchus* that digitally represents the musculoskeletal apparatus of its hindlimbs including joint ranges of motion (ROMs), body segment parameters (BSPs; e.g., body mass and center of mass; COM), and muscle moment arms (MMAs). We then use this model to aid in a critical review of whether *Postosuchus* was (1) quadrupedal or bipedal and (2) plantigrade or digitigrade, as two of our major questions (comparing models adopting these four poses). These two main questions involve exploring how musculoskeletal morphology correlates with those functions, including the usage of separate 3D models of *Postosuchus* with different plantigrade versus digitigrade bone articulations and comparison of their resulting ROMs. As a third major question, we investigate the functional and evolutionary implications of hindlimb muscle sizes estimated (via a volumetric digital model; e.g., Demuth et al., 2022; Herbst et al., 2022) for *Postosuchus*. We compare our estimates of muscle sizes with empirical data from an extant adult crocodile of similar body mass, but highly divergent morphology and function in numerous regards. We expect that, because *Postosuchus* and crocodiles differ considerably in locomotor morphology, especially with the former having a larger pelvis and hindlimb, the pelvic appendicular muscles of *Postosuchus* will be much larger: at least twice as massive relative to body mass. Answering this question enables tentative advances toward understanding how hindlimb muscle form and function varied in the few early archosauriforms and extant Crocodylia studied to date. That understanding will help build a future basis for reconstructing evolutionary trajectories in Archosauria, such as shifts in biomechanical specializations of hindlimb muscles with broader locomotor functions such as bipedalism.

## 2 MATERIALS AND METHODS

### 2.1 Photogrammetry and construction of composite skeleton

To capture photographs needed for photogrammetry, some bones (i.e., stylopodium, zeugopodium, autopodium, pelvis, ischia, ilium, scapulocoracoid, from TTU‐P 9000 and 9002) were placed horizontally on a turntable along with a scale and within a light tent integrating a dark background, which avoided light, motion, and position artifacts while preserving the original scale of the specimens. All phalanges from manus and pes respectively were all digitized together using one unique chunk of pictures. We ensured there was sufficient space between all phalanges in order to avoid overlapping in the resulting pictures while rotating the turntable. This process enabled the digitization of several small bones in just one 3D reconstruction for pes and manus, respectively. Pictures were taken using a Nikon D5500 or Nikon D40 camera placed on a tripod with 18–55 and 50 mm focal lenses depending on specimen size. Other bones (i.e., the vertebrae) were laid vertically on a rotating pad on which we stuck a graduated plastic rule for scale reference. We then set the pad on an unmovable 360° graduated plastic sheet in front of a vertical black background in order to avoid light reflection when shooting with a camera that was anchored on a tripod. During the shooting session, we manually rotated the pad with an increment of 10° in order to shoot every sample along a series of 36 photographs (under a 360° rotation), although the number of pictures varied based on number of views needed to capture the bone's shape and on the final quality of pictures. We repeated this protocol after laying the samples horizontally so that we obtained two chunks of ~36 pictures per bone for further full 3D reconstruction.

We 3D‐modelled each available bone that was used to reconstruct the skeleton of *Postosuchus kirkpatricki* via photogrammetric reconstruction using 3DF Zephyr 6.513 (https://www.3dflow.net/3df‐zephyr‐photogrammetry‐software/) and blender 2.92.0 (https://www.blender.org/); Agisoft Photoscan v1.0.3 (https://www.agisoft.com/; as in Clarac et al., 2017); and Metashape Professional v. 1.6.110009 and Meshlab v. 2020.06 (https://www.meshlab.net/; Cignoni et al., 2008; as in Pintore et al., 2022) software. For each bone, we created two 3D assemblages (one after each chunk), which we then exported in .STL format for further merging and scaling in Geomagic Wrap 2021 (https://oqton.com/geomagic‐wrap/). All 3D models were exported as .OBJ polygonal mesh files.

**FIGURE 2:**
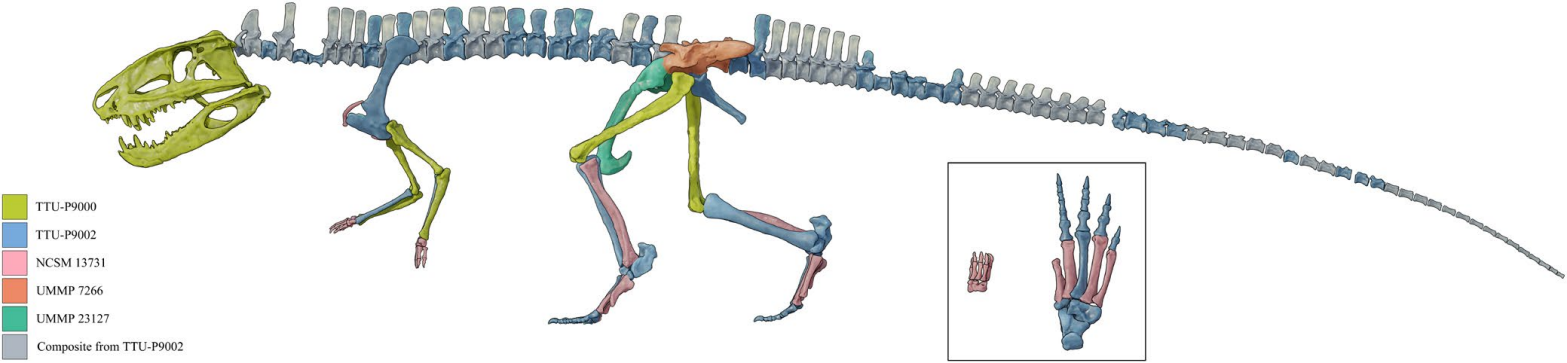
Skeletal elements of *Postosuchus kirkpatricki* used in the model, with skeleton reconstructed in a hypothetical digitigrade foot pose and bipedalism, in left lateral view. Color coding in the legend shows which bones came from which specimens, and dorsal (cranial) views of the left manus and pes are in the inset panel.

Table 1 shows what bones ultimately were used in the final model, and Figure 2 shows the composite skeletal reconstruction and which bones came from which specimens or were reconstructed. We favored TTU‐P 9000 as the focal specimen for our model because it is a large, presumably near‐adult specimen. The cast of the TTU‐P 9000 skull had been made by Jonathan Weinbaum, and a mesh file made from segmented CT scans of the cast was provided by Casey Holliday (University of Missouri). While we digitally smoothed most bone meshes, we did not correct for most taphonomic deformations. These were deemed modest for key elements in our biomechanical models, such as limb bones (see Chatterjee, 1985; Weinbaum, 2013). We also referred to the articulated left pes of NCSM 13731l for articulating the bones of that region. We used MT III from TTU‐P 9002, not NCSM 13731l, because we had good quality images for this, along with the phalanges, whereas MT III was too tightly “fused” in the digital model of NCSM 13731 that we had access to. We omitted pedal digit V's phalanges because their presence is unknown in *P. kirkpatricki*, and because they are tiny in *P. alisonae*, thus having miniscule effects on our analyses.

**TABLE 1:** Skeletal elements, specimens, and original image data used in the *Postosuchus kirkpatricki* model.

Skeletal elements	Specimen #(s)	# Images, resolution
Skull (cast)	TTU‐P 9000	CT scan of cast at University of Missouri School of Medicine, Department of Radiology (Siemens Somatom Definition Scanner, Siemens Medical Solutions USA Inc.), 3 mm slices
Atlas‐axis complex	N/A	Digitally sculpted based on Romer (1956, pp. 234–235)
Cervical centrum 3, cervical 5, 6 **[1.41]**	TTU‐P 9002	77 pictures, 3008 × 2000
Dorsal 2, 3, 10, 11 **[1.41]**	TTU‐P 9002	77 pictures, 3008 × 2000
Dorsal 6, 9, 12, 13 **[1.41]**	TTU‐P 9002	83 pictures, 3008 × 2000
Sacral vertebrae and ilium **[1.23]**	UMMP 7266	Original photogrammetry‐based mesh: 54,088 vertices, 107,553 polygons
Dorsal 15; Caudal 1, 2, 11 **[1.41]**	TTU‐P 9002	84 pictures, 3008 × 2000
Caudal 12, 13, 14, 15 **[1.41]**	TTU‐P 9002	79 pictures, 3008 × 2000
Caudal 23, 24, 25, 26, 27, 33, 36, 37, 38 **[1.41]**	TTU‐P 9002	84 pictures, 3008 × 2000
Clavicle **[1.04]**	NCSM 13731	Original photogrammetry‐based mesh: 90,008 vertices, 180,000 polygons
Interclavicle **[1.04]**	NCSM 13731	Original photogrammetry‐based mesh: 27,001 vertices, 53,998 polygons
Scapulocoracoid (left) **[1.41]**	TTU‐P 9002	137 pictures, 4496 × 3000
Humerus (right)	TTU‐P 9000	170 pictures, 4496 × 3000
Ulna (left)	TTU‐P 9000	36 pictures, 4496 × 3000
Radius (left) **[1.41]**	TTU‐P 9002	37 pictures, 4496 × 3000
Manus (right) **[1.04]**	NCSM 13731	Original photogrammetry‐based meshes: carpals = 13,821 vertices, 27,638 polygons https://www.morphosource.org/concern/parent/000678517/media/000678543; metacarpals and phalanges = 145,460 vertices, 290,900 polygons
Ischium **[1.41]**	TTU‐P 9002	168 pictures, 4496 × 3000, 300 dpi; 72 pictures, 3008 × 2000
Pubis **[1.08]**	UMMP 23127	Original photogrammetry‐based mesh: 23,156 vertices, 46,316 polygons
Femur (left)	TTU‐P 9000	168 pictures, 4496 × 3000, 300 dpi; 72 pictures, 3008 × 2000
Tibia (right) **[1.41]**	TTU‐P 9002	164 pictures, 4496 × 3000, 300 dpi; 156 pictures, 4496 × 3000, 300 dpi; 72 pictures, 3008 × 2000
Fibula (right) **[1.04]**	NCSM 13731	Original photogrammetry‐based mesh: 30,652 vertices, 61,300 polygons https://www.morphosource.org/concern/parent/000678517/media/000678593
Astragalus (left) **[1.41]**	TTU‐P 9002	41 pictures, 4496 × 3000
Calcaneum (left) **[1.41]**	TTU‐P 9002	31 pictures, 4496 × 3000
Metatarsals 1–2, 4–5 (left) **[0.963]**	NCSM 13731	Original photogrammetry‐based mesh: 631,339 vertices, 1,261,650 polygons
Metatarsal 3 (left) **[1.41]**	TTU‐P 9002	86 pictures, 3008 × 2000
Pes (left) **[1.41]**	TTU‐P 9002	86 pictures, 3008 × 2000, 300 dpi; 100 pictures, 4496 × 3000

*Note*: NCSM 13731 is *P. alisonae*. Numbers bracketed in bold font are scaling factors applied relative to TTU‐P 9000.

To reconstruct the vertebral column, 28 digitized vertebrae were photogrammetrically reconstructed using 3DF Zephyr 6.513 (see Table 1), then processed in blender 2.92.0. As not all vertebrae are known for *Postosuchus*, scaled reconstructions of 48 vertebrae were made using representative vertebrae from relevant sections of the column, referencing Romer (1956), Chatterjee (1985) and Weinbaum (2013) to ensure accuracy (also guided by Figure 1a). The distalmost caudal vertebra identified was caudal 38 (vs 30+ suggested by Chatterjee (1985)). All of the identified vertebrae were then placed corresponding to the skeletal reconstruction in Figure 1a. We adopted the estimated total of 53 caudal vertebrae from that reconstruction. Caudal vertebra 38 still has large enough articular processes that we deemed adding 15 decreasing caudals after it as reasonable. The overall appearance of the tail is quite gracile, but this seems real based on the reasonably well‐preserved caudals, including number 38. Any major damage or deformation in the photogrammetric reconstructions was digitally repaired, and the snake hook brush in blender was used to reconstruct the meshes to approximate their original morphology. Low and mid‐frequency details were then sculpted in blender to make accurate reconstructions of the missing vertebrae.

To address our questions about (1) quadrupedal versus bipedal and (2) plantigrade versus digitigrade poses in *Postosuchus*, we: (1) varied forelimb and hindlimb ROMs to explore what poses (and whole body center of mass positions relative to the base of support) would or would not enable quadrupedalism/bipedalism; and (2) constructed two models involving changes to the tarsal articulations representing different possible configurations (considered potentially plantigrade and digitigrade; e.g., Figure 1 vs Figure 2). Further details are in the Results and Discussion.

### 2.2 Design of jointed whole‐body model

Our protocol for model construction followed that first outlined by Bishop, Cuff, et al. (2021) and followed by our subsequent studies (Bishop, Falisse, et al., 2021, Bishop, Michel, et al., 2021; Demuth et al., 2020, 2023; Lecuona et al., 2025; Otero et al., 2024; von Baczko et al., 2025; Wiseman et al., 2021). We first built a 3D skeletal model (“digital marionette”), as follows. Bone mesh files (.OBJ format) were decimated to <50,000 polygons in MeshLab. We initially articulated the bones into a “reference pose” (sometimes termed “neutral posture”), with vertebrae positioned in the approximate middle of intervertebral joint contacts, and the limbs posed vertically in a digitigrade orientation (see Results and Discussion for more exploration of this approach, and comparison with a plantigrade variant). Manus and pes interphalangeal joints were not modelled, and the distal autopodium solely used the third metapodial bones to position the bases of the digits, so the manus and pes digits all respectively moved together. We digitally isolated the articular surfaces of the joints by editing the mesh files in MeshLab. Intervertebral joints were formed between centra of the intervertebral joints at the 7th–8th (“neck”) and 19–20th (“back”) trunk vertebrae, and the 2nd and 3rd (“proximal tail”) and 24th–25th caudal vertebrae (“distal tail”); in the middle of the tail; as well as using the two sacral vertebrae to form the craniocaudal axis of the model. These intervertebral joints were formulated using the isolated ventral to lateral surfaces (with each centrum a “U” shape in cross‐section) of adjacent centra. We used an analogous procedure to estimate the articular surfaces of the (forelimb) glenoid and proximal and distal humerus, radius and ulna, and third metacarpal; and (hindlimb) acetabulum, femur, tibia and fibula, proximal tarsals, and third metatarsal. We used the MATLAB 2024 (The MathWorks, Inc., Natick, MA, USA) scripts from Bishop, Cuff, et al. (2021) to automatically fit 3D geometric primitives to those joint surfaces, with shapes chosen as cylinders for the vertebrae and distal ends of all limb bones. Exceptions to cylinders were: spheres for the glenoid, proximal humerus, proximal and distal radius and ulna, and acetabulum; an ellipsoid for the proximal femur; and planes for the proximal ends of other limb bones (except radius and ulna). We then exported these primitives' OBJ meshes and imported them into Rhinoceros 7.0 (McNeel and Associates, Barcelona, Spain) software to complete the subsequent modelling steps.

**FIGURE 3:**
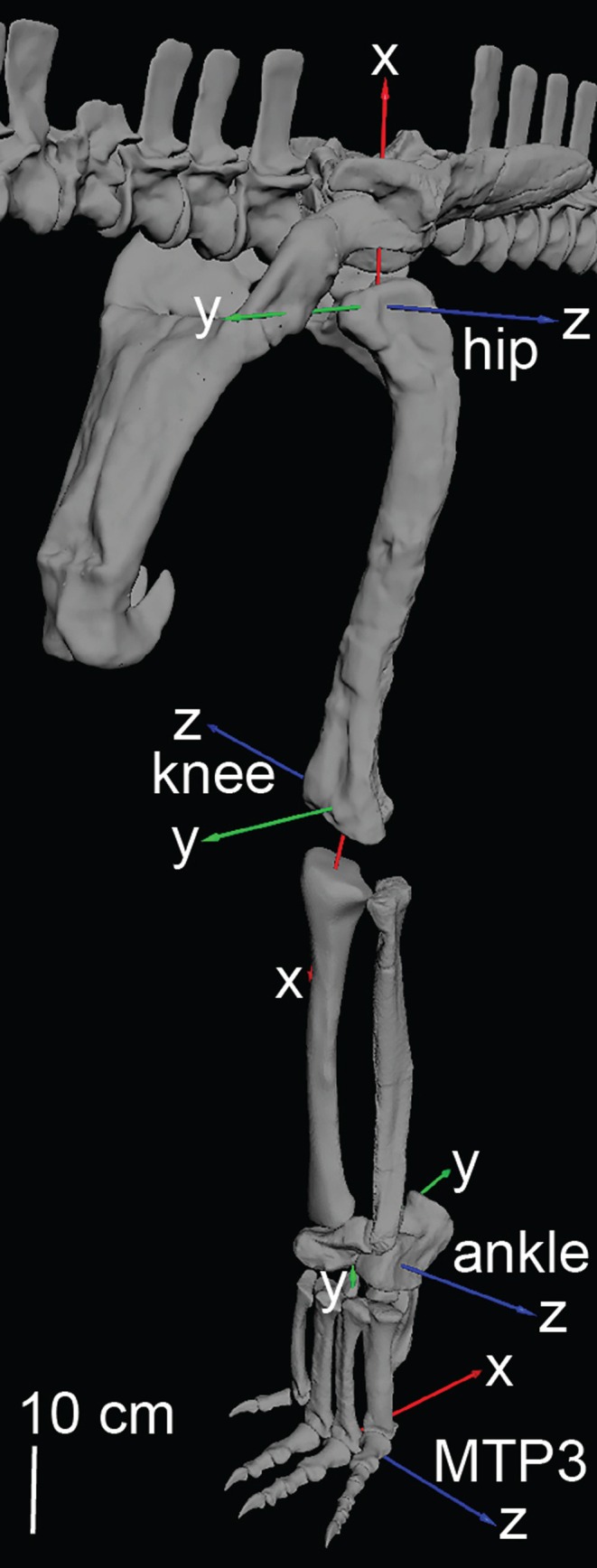
Left hindlimb joint coordinate systems (JCSs) for the *Postosuchus* model, in oblique craniolateral view. Joints (hip, knee, ankle, and MTP3 = third metatarsophalangeal) are labelled next to their flexion/extension axes. Red, green, and blue colored axes (*x*, *y*, *z*, respectively) are external/internal long‐axis rotation, abduction/adduction, and extension/flexion as labelled (following Gatesy et al., 2022). The limb is in the reference pose (all angles = 0°). Arrows point toward positive values of axes. The ankle joint's *x*‐axis is hidden beneath the metatarsus (as is most of the MTP3's *y*‐axis).

We used the geometric primitives to define anatomical coordinate systems (ACSs) as per Bishop, Cuff, et al. (2021) and Gatesy et al. (2022) and references therein, maintaining right‐handed ACSs (*x*, *y*, *z* vectors). This process then paired ACSs to define joint coordinate systems (JCSs); again following Bishop, Cuff, et al. (2021) and Gatesy et al. (2022); depicted in Figure 3 for the hindlimb and Figure S1 for the forelimb (intervertebral JCSs are not shown but followed Bishop, Cuff, et al., 2021, Bishop, Falisse, et al., 2021, Bishop, Michel, et al., 2021). Where necessary, we adjusted bone and JCS positions to maintain the reference pose values of joints as close to 0° as possible (e.g., parasagittally straight proximal limb segments) and minimize bone overlap. Joints rotated following a *z*, *y*, *x* axis rotation order. As explained by Lecuona et al. (2025), positive/negative angles of limb joints had external/internal long‐axis rotation (LAR) about the JCS's *x*‐axis; abduction/adduction about the *y*‐axis; and extension/flexion about the z‐axis for the hip, third metatarsophalangeal (MTP3), shoulder and third metacarpophalangeal (MCP3) joints. The x, y, and z axes' positive values for the knee were: internal LAR, abduction and extension (solely the latter allowed in the model); for the ankle: internal LAR, adduction, extension (solely the latter allowed in the model), for the elbow were: internal LAR, abduction, flexion; and for the wrist: internal LAR, abduction and extension (solely the latter allowed in the model; palmarflexion) (Lecuona et al., 2025). Limb joints had one degree of freedom (DOF) for all except for 3 DOF for the hip and shoulder joints, and allowing elbow abduction/adduction (allowed because the joint surfaces imply more than hinge‐like motion; but not otherwise used in this study). The proximal humerus and femur's ACSs were respectively matched to the glenoid and acetabulum ACSs, so the shoulder and hip joint centers were at the middle of those spaces. We translated the forearm and crural bones and JCSs distally by 10% of their length (i.e., space added between distal humerus/femur and proximal radius‐ulna/tibia‐fibula) to add space for thick articular cartilage (Holliday et al., 2010), adhering to our other studies' standards (Bishop, Cuff, et al., 2021; Demuth et al., 2020; Hutchinson et al., 2005). The tightly fitting radioulnar‐proximal carpal, intertarsal and metacarpophalangeal and metatarsophalangeal joints had no added space, whereas we lacked bone geometry for the distal carpals and thus added a proximodistal space between the proximal carpals and metacarpals approximating that small distance. We finalized JCSs in Autodesk Maya (San Francisco, California, USA) with ACSs incorporated (Brainerd et al., 2010; Gatesy et al., 2022) using the XROMM_MayaTools plugin (https://bitbucket.org/xromm/xromm_mayatools/src/master/); then JCSs were exported as .OBJ files for usage in a MATLAB 2024 script Bishop, Cuff, et al. (2021) based on that produced a 3D musculoskeletal model for further analyses (see below).

### 2.3 Joint morphology and ranges of motion

As we explain in the Results and Discussion, we used the digital marionette and its underlying skeletal morphology to qualitatively explore what limb poses (plantigrade/digitigrade; quadrupedal/bipedal) were plausible. We also quantitatively estimated limb joint ranges of motion (ROMs) one DOF at a time (as followed by our earlier studies such as Bishop, Cuff, et al., 2021; Lecuona et al., 2025; Otero et al., 2017, 2024; Pierce et al., 2012; von Baczko et al., 2025). Our model requires ROM limits to be specified or else all joints have extremely unrealistic 360° ROMs. The ROMs we obtained enable straightforward comparisons with the latter studies' ROM estimates. Our simple manual approach approximated excessive disarticulations or bone collisions as ROM limits, with 5° increments. Such ROM estimation incurs problematic assumptions such as omitting interactions of DOFs and the absence of joint translations, which can make ROMs misleading relative to more detailed “joint mobility” hypervolumes (Manafzadeh & Gatesy, 2021, 2022), although sometimes there are qualitative correspondences (Brocklehurst et al., 2022; Regnault et al., 2021). While these ROMs are flawed, they and our model (which can be modified to add more complex joints) form a reasonable basis for future studies to expand upon (e.g., Manafzadeh et al., 2024). We did not investigate intervertebral ROMs here but our model assumed roughly reasonable ROMs of −30° to 30° dorsiflexion and lateroflexion (Bishop, Michel, et al., 2021).

### 2.4 Estimating body segment parameters of the model

To transform the skeletal model into a model of the whole *Postosuchus* organism, we adopted the same methods we have used in prior work (Hutchinson et al., 2007, 2011, Allen et al., 2009, 2013 and other studies), detailed by Bishop, Cuff, et al. (2021). This transformation produced a reconstruction of the 3D body shape of *Postosuchus* so that we could use those dimensions to compute segmental mass (inertial) properties—or body segment parameters (BSPs)—needed for biomechanical analyses. This proceeded by 10 main steps in Rhinoceros software: (1) Fitting octagonal polygonal hoops sequentially along the longitudinal axis for each major body segment craniocaudally (axial segments) or proximodistally (limb segments). The axial segments were the head and neck, front and back halves of the torso (united by the “back” joint), “body” (pelvis‐sacrum region), proximal tail, and distal tail. Forelimb segments were the upper arm, forearm, manus, and manual digits. Hindlimb segments were the thigh, crus/shank, pes, and pedal digits. (2) Air cavities were constructed via an identical octagon‐based polygonal hoop‐building approach representing zero‐density volumes in the cranium (pharynx, sinuses), neck (trachea), and thorax (lungs). (3) The initial hoops closely followed skeletal outlines where possible. (4) However, in many regions, it was necessary to use a different approach. Because the upper arm and thigh should have been considerably larger than the proximal humerus or femur, we expanded those regions to more realistically converge on the pectoral/pelvic girdles where muscles would have originated. (5) We lacked any scan data for ribs, gastralia, and caudal chevrons/haemal arches in particular. The body shape in these regions was estimated by, for the trunk, fitting an ellipse to the internal boundaries of the vertebral column and girdles, and, for the tail, by expanding the 3D boundaries from the starting shape following shapes of extant saurian tails from Allen et al. (2009), and where necessary by using the reconstruction in Figure 1. (6) Our overall approach still was expected to underestimate body dimensions (e.g., Allen et al., 2009, 2013; Bishop, Cuff, et al., 2021; Hutchinson et al., 2007, 2011). Thus, we expanded all octagonal hoops by set factors (see Otero et al., 2024 for examples) and lofted them together to create mean 3D body segment shapes. (7) Limb segments were mirrored left‐to‐right to ensure a bilaterally symmetrical model, and all shapes were exported from Rhinoceros as .OBJ meshes. (8) All .OBJ meshes were converted into all‐triangular meshes in Meshlab, and placed into the same coordinate system as the Maya “digital marionette” for creation of a musculoskeletal model. (9) The “mass” shapes were given densities of 1060 kg m^−3^ (Méndez & Keys, 1960; Ward & Lieber, 2005) but the air cavities reduced the actual segment densities. (10) Finally, we used custom MATLAB scripts from Bishop, Cuff, et al. (2021) to convert meshes oriented in and exported from the Maya model to an OpenSim (version 4.1) .osim format model (https://simtk.org/projects/opensim; Seth et al., 2018; see below). These scripts included formulation of BSPs (masses and centers of mass; inertial tensors not dealt with here) for the model, also enabling expression of the whole‐body center of mass (COM). A more sophisticated version from Strong et al. (2025) was deemed unnecessary for our purposes.

Our new model had BSPs and other dimensions that deserved comparison to prior estimates and analyses, to check its impact on predictions about locomotor function. We input the body mass, COM (made dimensionless by dividing by gleno‐acetabular distance), and forelimb and hindlimb lengths from our model into Bishop et al.'s (2020) dataset for models of 80 archosauriforms. They used Henderson and Snively's (2004) 3D model of *Postosuchus* in a statistical, morphometric analysis of body dimensions. We repeated Bishop et al.'s (2020) linear discriminant analysis (LDA) using our modified data in the MASS package for R (v. 7.3‐50; Venables & Ripley, 2002). See Bishop et al. (2020) for full details of the pPCA methodology. Briefly, the LDA conducted 22 analyses of training datasets that predicted bipedalism versus quadrupedalism for taxa with “unknown” stances as compared with the broader dataset of “known” bipeds and quadrupeds. Additionally, Spiekman et al. (2024) provided a dataset of humeral and femoral circumferences for a variety of known quadrupeds and bipeds, with R code for estimating the stance of the “sphenosuchian” (early crocodylomorph) *Terrestrisuchus*, finding that it mostly likely was quadrupedal. We added our data for *Postosuchus* and reran that analysis to test how it compared to the bipedal and quadrupedal morphospaces.

### 2.5 Creation of the musculoskeletal model

**FIGURE 4:**
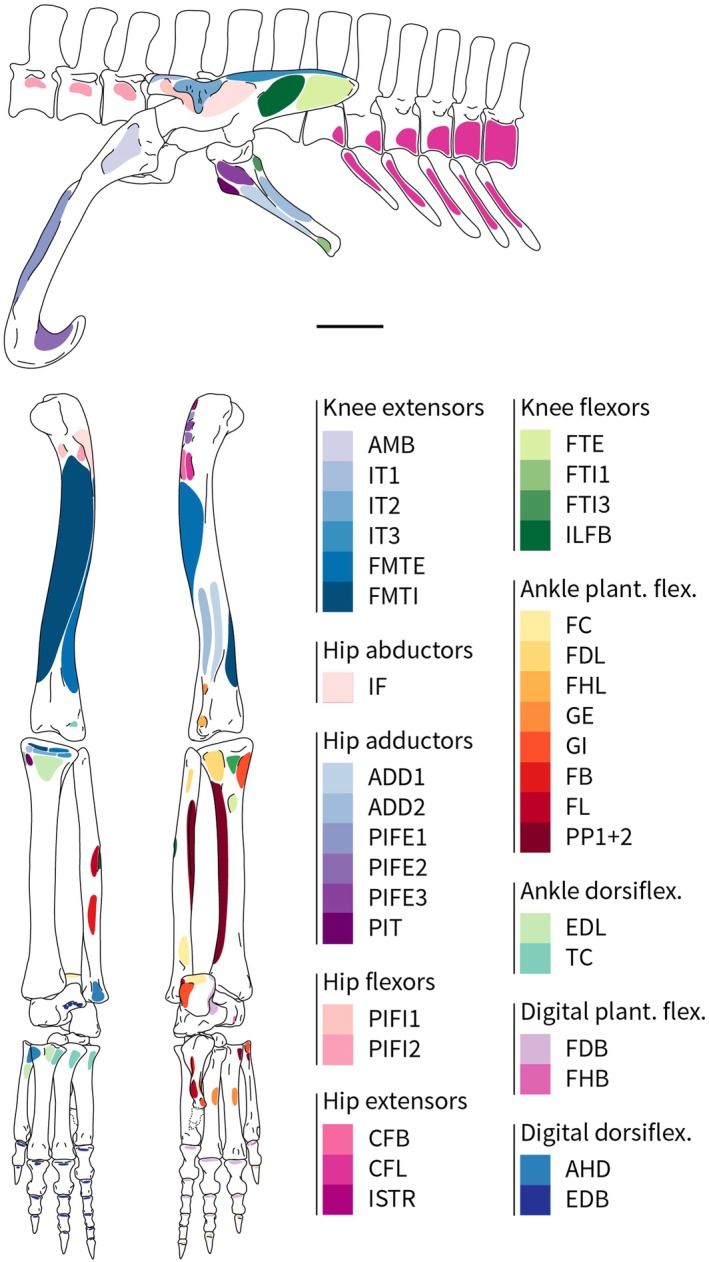
“Muscle map” illustrating left hindlimb musculature reconstructed for *Postosuchus*, used for musculoskeletal modelling including volumetric muscle reconstruction. Hindlimb is fully extended. Shown: Top, pelvis and nearby vertebrae with muscle origins. Only parts of the PIFI2 and CFL muscle origins are depicted; other attachments such as CFB origin are not visible. Bottom left, hindlimb in cranial view with muscle origins and insertions. Bottom right, hindlimb in caudal view with muscle origins and insertions. See Table 2 for acronyms. Legend shows color coding for muscle acronyms (in Table 2), organized by rough functional groups (“plant. flex.” = plantarflexors; “dorsiflex” = dorsiflexors). Scale bar is 10 cm.

**FIGURE 5:**
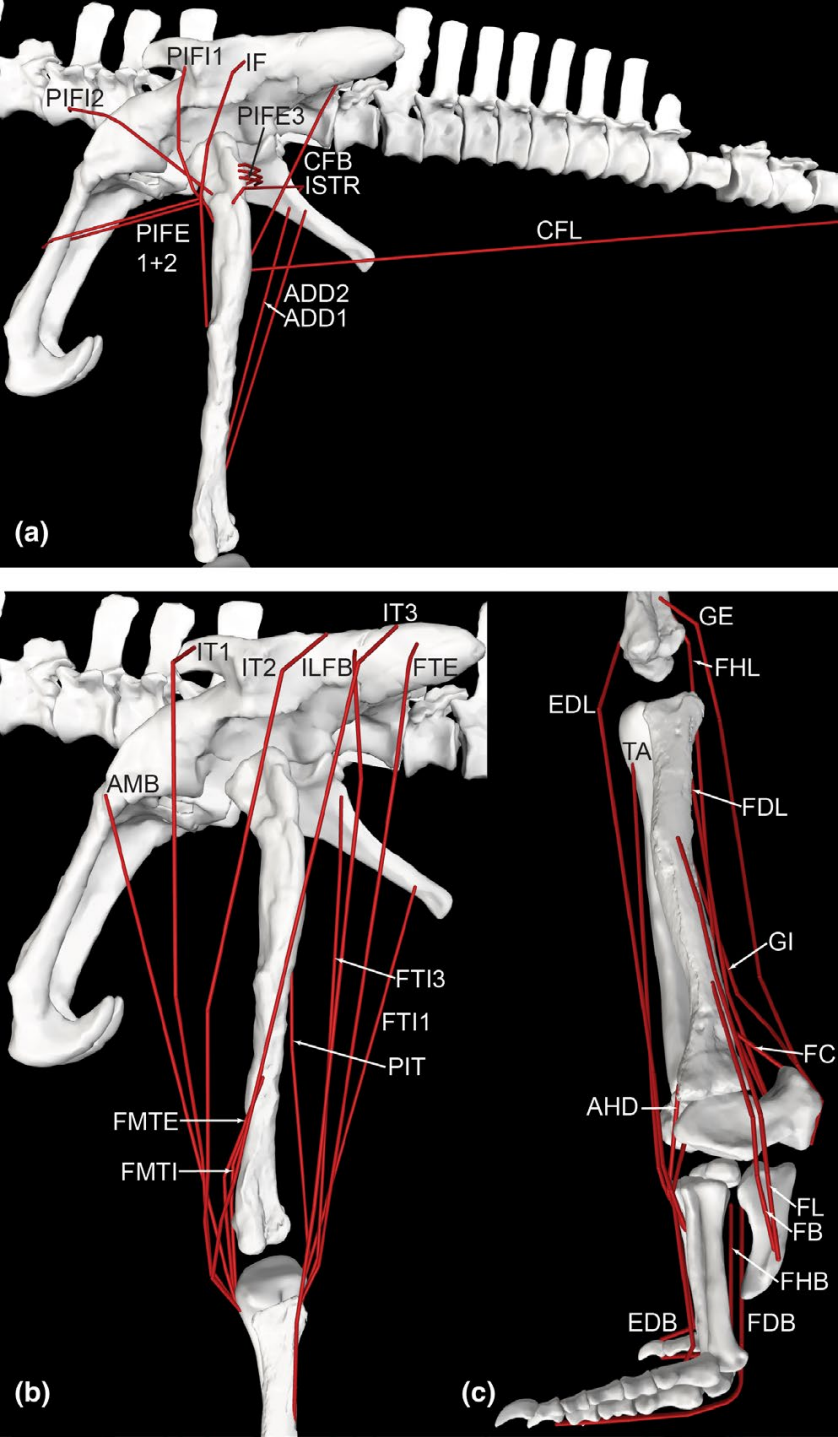
Left hindlimb musculature reconstructed in OpenSim for *Postosuchus*, in lateral views. (a) major deep hip flexors and extensors. (b) “Triceps femoris” knee extensors and “hamstring” hip extensors/knee flexors. (c) lower limb muscles (PP1, PP2, and EHL not visible). See Table 2 for acronyms. Not to scale.

3D paths of hindlimb muscles in *Postosuchus* were based on phylogenetic character mapping of osteological correlates of muscle attachments across Sauria; using the Extant Phylogenetic Bracket (EPB; Witmer, 1995); with a modified character matrix from Hutchinson (2001a, 2001b, 2002) and Bishop, Cuff, et al. (2021); provided in the Supporting Information. We traced character states for 107 characters of 48 taxa using maximum parsimony optimization in Mesquite 3.81 software (Maddison & Maddison, 2023; http://www.mesquiteproject.org). This process inferred character states for muscle origins and insertions in *Postosuchus* (Table 2), using visible osteological correlates of those soft tissue attachments. Only non‐ambiguous states were taken from the tree, and then interpreted in light of the EPB method. We added these states to our OpenSim model, representing approximate centroids of muscle origins, insertions, and 3D paths constrained by “via points” and “wrapping surfaces” (see Allen et al., 2021; Bishop, Cuff, et al., 2021; Hutchinson et al., 2005, 2015), and adjusting paths so that they remained anatomically realistic throughout joint ROMs. We omitted muscles that did not act around any DOFs in our model; for example, most muscles of the pes; and because their mechanical influences remain not completely tested, we omitted the secondary tendons of thigh muscles such as the CFL and AMB that might transmit forces around the ankle (Ito et al., 2024). M. extensor hallucis longus (EHL) was reconstructed only in the musculoskeletal model, for completeness and because it could actuate the metatarsophalangeal joint of digit I if that joint was made mobile (easily modified using our model). Note that it was omitted from the models of Lecuona et al. (2025) and Otero et al. (2024) but included in von Baczko et al. (2025). We created a digitigrade musculoskeletal model and then a plantigrade one that differed in bone articulations and some minor details of muscle paths (mainly to avoid cutting through bones in the plantigrade poses; and to properly attach to bones that were moved). Figures 4 and 5 show the final model. We used custom MATLAB code (from Lars d'Hondt, KU‐Leuven, Belgium) to mirror the model from left to right side, ensuring symmetry. For comparing MMAs around the ankle and metatarsophalangeal joints in plantigrade versus digitigrade model configurations, we exported plots of MMAs versus joint angles across those joints' ROMs and computed mean and median MMAs for each muscle, and ratios of those MMAs for plantigrade versus digitigrade models.

**TABLE 2:** *Postosuchus* pelvic limb muscle origins and insertions reconstructed here.

Muscle	Origin	Insertion
M. iliotibialis 1 (IT1)	Craniodorsal iliac rim (roughening) [I]	Cranial tip of cnemial crest of tibia [I]
M. iliotibialis 2 (IT2)	Dorsal iliac rim and extending ventrally onto supra‐acetabular buttress[I]	Cranial tip of cnemial crest of tibia [I]
M. iliotibialis 3 (IT3)	Caudodorsal iliac rim (roughening) [I]	Cranial tip of cnemial crest of tibia [I]
M. femorotibialis externus (FMTE)	Lateral femoral shaft, between intermuscular lines and limited proximally by PIFI2 insertion [I]	Cnemial crest of tibia [I]
M. femorotibialis internus (FMTI)	Medial femoral shaft, between intermuscular lines, limited proximally by PIFI1 and CFL insertions [I]	Cnemial crest of tibia [I]
M. ambiens (AMB)	Pubic tubercle of proximal pubis [I]	Cnemial crest of tibia [I]
M. iliofibularis (ILFB)	Lateral surface of postacetabular ilium, between IF and FTE [I´]	Iliofibular tubercle/scar on craniolateral fibular mid‐shaft [I]
M. iliofemoralis (IF)	Lateral surface of ilium above acetabulum (depression; Chatterjee, 1985) [I]	Caudolateral side of femoral mid‐shaft (knob) [II]
M. puboischiofemoralis internus 1 (PIFI1)	Ventrolateral side of preacetabular ilium (and possibly from medial side of pelvis) [II]	Craniomedial proximal femoral shaft, lateral to fourth trochanter [I´]
M. puboischiofemoralis internus 2 (PIFI2)	“Lumbar” (dorsal) vertebrae close to preacetabular ilium; surfaces of centra laterally and transverse processes ventrally [II]	Craniolateral proximal femur (scar on craniolateral side of proximal femur) [I]
M. puboischiotibialis (PIT)	PIT (one head) on craniolateral proximal ischial apron, craniad to other ischial muscles [II]	Medial (extending to caudal) proximal tibia; depression in *P. alisonae* (Peyer et al., 2008) [I]
M. flexor tibialis internus 1 (FTI1)	Lateral surface of distal ischial shaft [II´]	Medial (extending to caudal) proximal tibia; depression in *P. alisonae* (Peyer et al., 2008) [I]
M. flexor tibialis internus 3 (FTI3)	Proximolateral ischial tuberosity [I´]	Caudal (extending to lateral) proximal tibia [I´]
M. flexor tibialis externus (FTE)	Lateral surface of caudoventral corner of postacetabular ilium [I´]	Caudal (extending to lateral) proximal tibia [I´]
M. puboischiofemoralis externus 1 (PIFE1)	Cranial surface of pubic apron [II]	Greater trochanter of femur [I]
M. puboischiofemoralis externus 2 (PIFE2)	Caudal surface of pubic apron [II]	Greater trochanter of femur [I]
M. puboischiofemoralis externus 3 (PIFE3)	Lateral surface of ischial apron, caudal to ADD1 [II´]	Greater trochanter of femur [I]
M. ischiotrochantericus (ISTR)	Medial surface of ischial apron [I]	Lateral side of proximal‐most femur, near PIFE1–3 [I´]
M. caudofemoralis brevis (CFB)	“Brevis” fossa of ilium, and sacral/proximal caudal vertebrae [I]	Caudolateral side of proximal fourth trochanter [I]
M. caudofemoralis longus (CFL)	Lateral surfaces of haemal arches/chevrons and transverse processes of proximal caudal vertebrae [I]	Fourth trochanter of femur; medial pit [I]
M. adductor femoris 1 (ADD1)	Craniolateral surface of ischial apron and shaft; cranial to PIFE3 (cranial to a longitudinal ridge; Chatterjee, 1985; Weinbaum, 2013) [I´]	Caudomedial distal femoral shaft [I´]
M. adductor femoris 2 (ADD2)	Caudolateral surface of dorsal ischial shaft, from scarred groove (somewhat evident in TTU‐P 9002; clearer in DMNH 27724) [I]	Caudolateral distal femoral shaft near caudal intermuscular line [I´]
M. gastrocnemius internus (GI)	Medial side of cnemial crest of proximal tibia [I´]	Dorsal end of calcaneal tuber and plantar aponeurosis to metatarsal V (groove), process on distal tarsal IV, and metatarsals II + III, then to digits 2–4 with FDB [II]
M. gastrocnemius externus (GE)	Caudolateral distal femur, proximal to lateral condyle [I´]	Dorsal end of calcaneal tuber and plantar aponeurosis (groove on tuber), then to metatarsal V [II]
M. extensor digitorum longus (EDL)	Lateral side of the cnemial crest; distal to TA origin [II]; and the cranial tibial shaft [I]	Craniomedial surfaces of proximal metatarsals I and II [I´]
M. extensor digitorum brevis (EDB)	Cranial surfaces of proximal tarsals [II´]	Dorsal surfaces of distal phalanges [I]
M. extensor hallucis longus (EHL)	Cranial surface of proximal metatarsal I [I]	Dorsal surfaces of digit 1 phalanges [I]
M. tibialis anterior (TA)	Craniolateral side of the distal femur [I], and lateral side of cnemial crest [II]	Craniomedial sides of proximal metatarsals II–IV [I´]
M. flexor digitorum longus (FDL)	Proximomedial fibular shaft [I´]	Flexor tubercles of pedal unguals II–IV [I]
M. flexor hallucis longus (FHL)	Caudolateral distal femur near GE origin, cnemial crest of tibia, fossa flexoria, and proximal fibula; craniolateral scar on tibia in *P. alisonae* (Peyer et al., 2008) [I]	Flexor tubercles of pedal unguals I–IV [I]
M. flexor digitorum brevis (FDB)	Plantar aponeurosis [I′]	Flexor tubercles of pedal unguals I–IV [I]
M. flexor hallucis brevis (FHB)	Distal tarsals and plantar aponeurosis [I´]	Plantar surfaces of metatarsal I and digit 1, first phalanx [I′]
M. fibularis longus (FL)	Lateral shaft of fibula, distal to ILFB insertion [I´]	Lateral side of metatarsal V [I]; and calcaneal tuber [II]
M. fibularis brevis (FB)	Distalmost craniolateral shaft of fibula, distal to FL origin [I´]	Caudolateral side of metatarsal V (and IV); proximal to FL [I]
M. interosseous cruris/proximal pronator profundus (PP1)	Caudolateral proximal tibial shaft [I´]	Caudolateral side of metatarsal I (and II–III) and tarsals (especially process of distal tarsal IV) [I]
M. pronator profundus (PP2)	Caudomedial fibular shaft [I´]	Caudolateral side of metatarsal I (and II–III) and tarsals (especially process of distal tarsal IV) [I]
M. fibulocalcaneus (FC)	Caudal fibular surface, distal fourth [I´]	Dorsal (proximal) surface of calcaneal tuber [II]
M. abductor hallucis dorsalis (AHD)	Craniolateral side of distal fibula [I´]	Proximodorsal (cranial) surface of metatarsal I, near EDL insertion [I]

*Note*: Names [and acronyms] follow those conventionally used for Crocodylia (Cong et al., 1998; Hattori & Tsuihiji, 2021; Hutchinson, 2002; Wilhite, 2023; Pereyra et al., 2024; Romer, 1923a, 1923b). Origins and insertions include levels of inference (Witmer, 1995) in []: I = unequivocal; II = equivocal; with ´ denoting absence of a clear osteological correlate. Only muscles included in the musculoskeletal model are listed, often with simplified attachments because each muscle only had a single line of action (no branching origins or insertions), such as those from thigh muscles to the crus. This format closely follows our prior publications' (e.g., Bishop, Cuff, et al., 2021; Lecuona et al., 2025; Otero et al., 2024; von Baczko et al., 2025).

### 2.6 Volumetric model of hindlimb musculature

**FIGURE 6:**
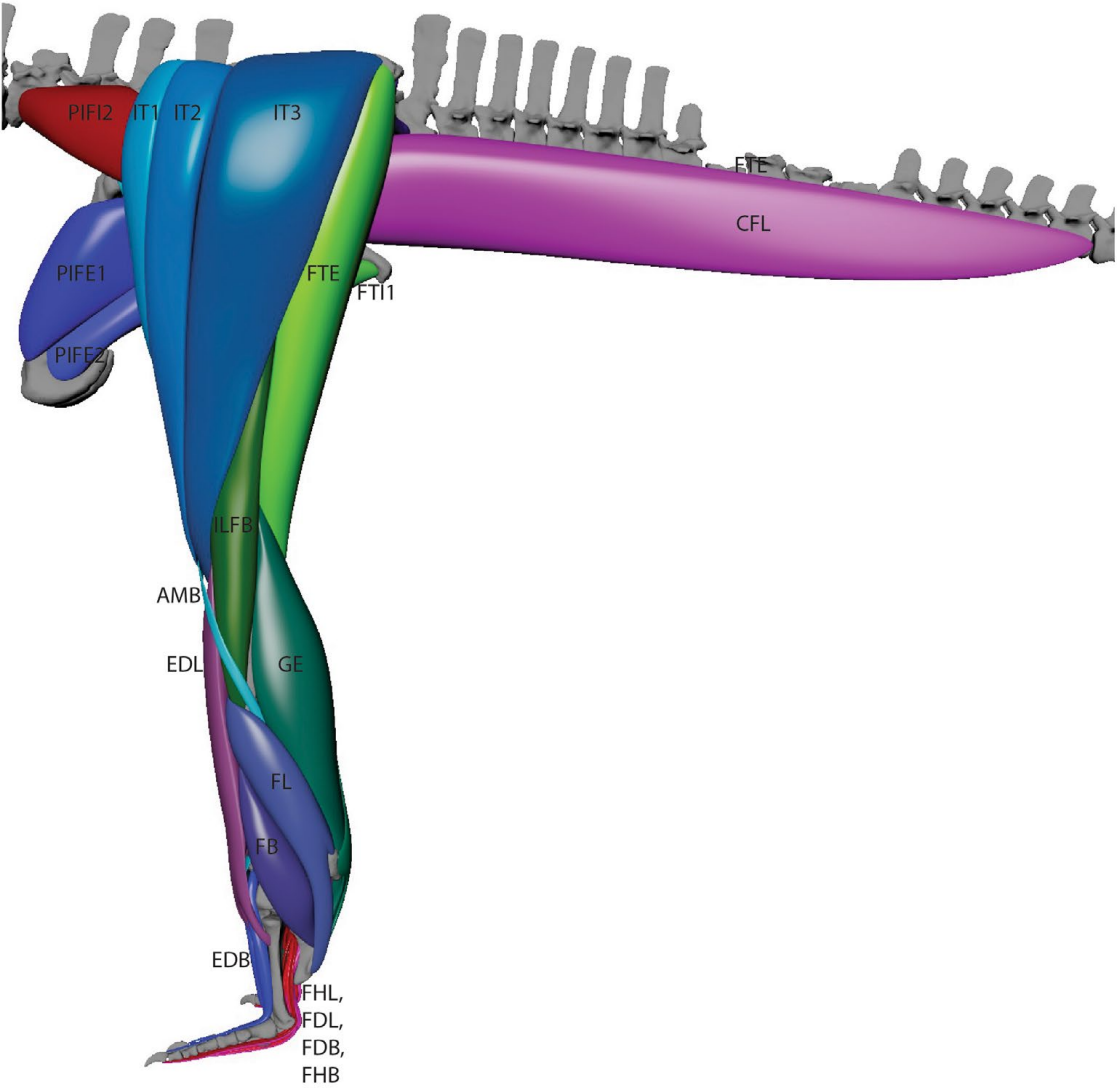
Volumetric reconstructions of left hindlimb muscles in *Postosuchus*, in lateral view. Only the most superficial muscles are shown; see Figures 4 and 5 for further detail; see Data Availability for the complete set of muscle files. See Table 2 for acronyms. Not to scale.

**FIGURE 7:**
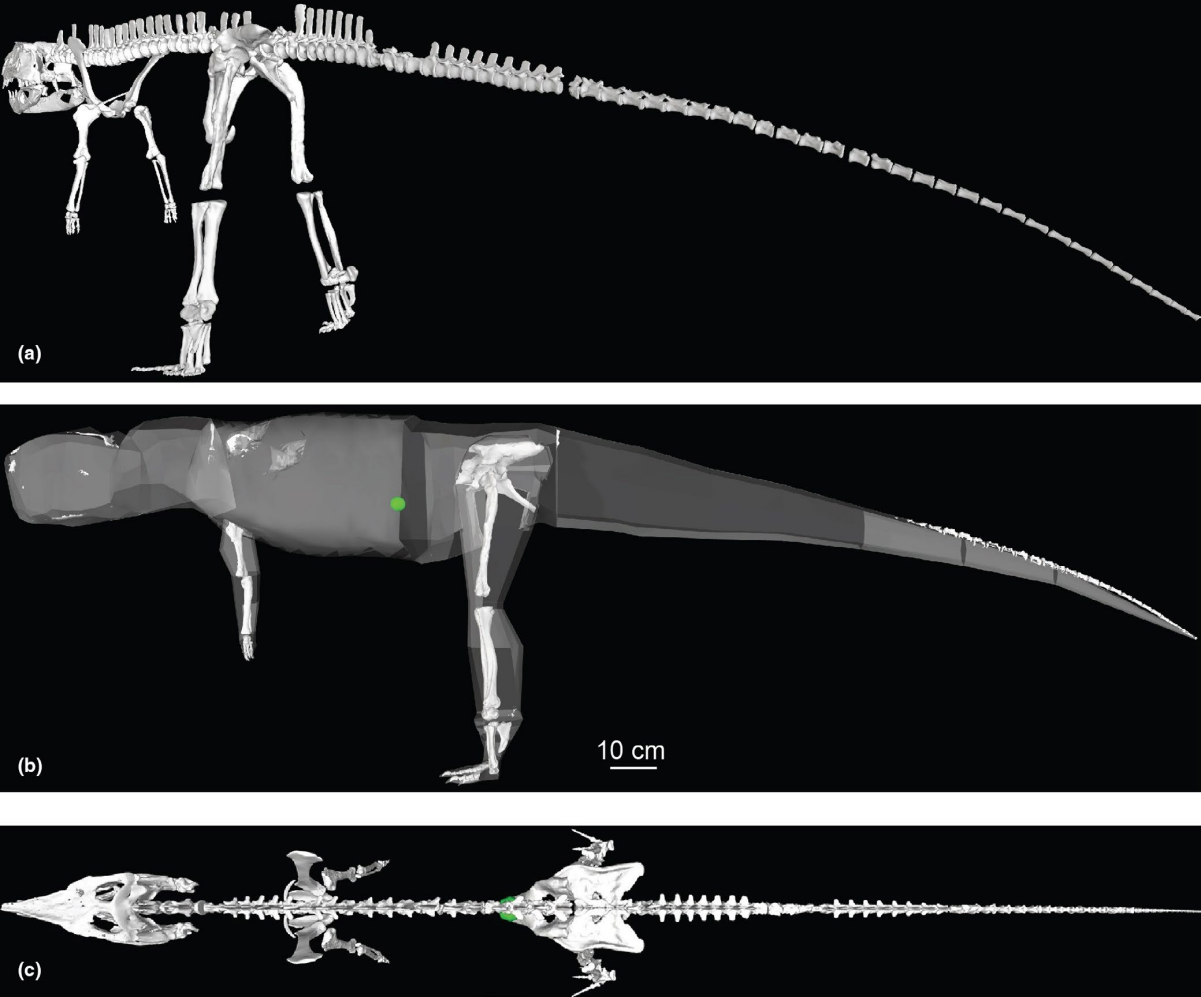
Whole‐body model of *Postosuchus*. (a) In left caudolateral view, showing bones as per Table 1. Forelimbs and hindlimbs have been abducted by 15° from the reference pose at 0°. (b) In left lateral view, with transparent objects representing segment shapes used to calculate BSPs (only showing left side); and whole‐body COM (green sphere). (c) In dorsal view of the skeleton, showing the more caudal COM position (green sphere near pelvis) when the head and neck mass was decreased and the proximal tail mass was increased (see text).

In addition to our OpenSim musculoskeletal model, we used the volumetric muscle modelling method of Demuth et al. (2022) to estimate hindlimb muscle masses in *Postosuchus*. This involved adding the muscles to the Maya model that was used to add ACSs and JCSs to the model (Figure 3), and hence the muscles were in the same 3D coordinate system as the OpenSim model. We added muscles first by using Maya's *Quad Draw* function of the *Modelling Toolkit* to trace the origins (and later, insertions) onto the surfaces of the bone meshes, then extruded these polygons toward the insertions, with scaling, rotation, and translation to adjust muscle cross‐sectional shapes. Once complete, muscles were iteratively sculpted into smooth shapes that fit with all other objects (bones and muscles) in the Maya file (Figure 6). To help guide muscle placements, as in Demuth et al. (2022), we placed 2D cross‐sections of *Alligator mississippiensis* hindlimbs (see Wilhite, 2023) that included all muscles into the Maya file, scaled to match the perimeters of the body segments (Figure 7a). Some muscles of the pes were not sufficiently clear in the 2D cross‐sections, so we used Demuth et al.'s (2022) *Euparkeria capensis* muscles to guide these (*Euparkeria* is a close outgroup to Archosauria; Figure 1 and Nesbitt, 2011). Each muscle volume then was processed through a custom Maya script (Demuth et al., 2022; modified from Allen et al., 2017) to create a line fit through the centroid of each cross‐section of that volume, producing a .OBJ mesh exported and automatically added as a “body” (object linked to a body segment, such as the femur) into the OpenSim model. Muscles with multiple origins and insertions only used the major (largest; or best representing the overall line of action of the muscle) attachments to constrain their paths. We then adjusted our initial OpenSim muscle paths to match these lines while keeping the lines smooth and constrained by the via points and wrapping surfaces (final paths are in Figures 4 and 5). We did not vary the volumetric muscle model to adopt a different myology in the plantigrade pose; here we purely focus on results from the digitigrade model. Muscle volume values were automatically calculated by code from Demuth et al. (2022), and converted into masses by assuming a standard vertebrate striated muscle density of 1060 kg m^−3^ (Hutchinson et al., 2015; Méndez & Keys, 1960; Ward & Lieber, 2005). To place our estimates in a comparative context versus empirical data from an extant archosaur of comparable size, we compiled the muscle‐tendon unit masses from a 278 kg Nile crocodile (*Crocodylus niloticus*) from the dissection‐based muscle architecture data in Allen et al. (2014). Comparisons were made using muscle masses as percentages of body mass and of total hindlimb muscle mass.

## 3 RESULTS AND DISCUSSION

First here we describe aspects of hindlimb myology reconstructed for *Postosuchus* and compare them with the literature. Second, we present the model's BSPs, how they relate to estimates from other methods and modelling assumptions, and what these new calculations suggest about quadrupedalism/bipedalism. Third, we consider how our estimated hindlimb ROMs compare with joint morphology, and thereby what aspects of hindlimb joint function in *Postosuchus* are plesiomorphic versus derived. Fourth, we explore in depth what evidence from our model and other potential data (e.g., ichnological; pes proportions), as well as a second model adjusted into a plantigrade conformation, supports inferences about plantigrade versus digitigrade pes stance in *Postosuchus*. Fifth, we compare different lines of evidence from our model (BSPs, manus form and function, and joint ROMs) regarding quadrupedalism versus bipedalism in *Postosuchus*. Finally, we present our results from estimating hindlimb muscle masses, comparing these values to an extant crocodile, and how they relate to plesiomorphic versus derived hindlimb function in *Postosuchus*.

### 3.1 Hindlimb myology

Figure 4 shows a “muscle map” for hindlimb muscle attachments used in our model of *Postosuchus* (Table 2), and elaborated on to estimate muscle masses later in our study. Figure 5 shows the resulting 3D musculoskeletal model, and Figure 6 contains a view of the 3D volumetric muscle model. More explanation of differences between our general findings for this suchian archosaur versus others in the literature (e.g., Bates & Schachner, 2012; Liparini & Schultz, 2013; Schachner et al., 2011; Walker, 1964, 1970) was provided by von Baczko et al. (2025) and we do not repeat them here, especially as most (except perhaps for those detailed above) would likely have small functional implications.

Some muscle scars that are typical of archosaurs (Hutchinson, 2001a) are evident, such as the M. ambiens (AMB) origin from the lateral side of the proximal pubis, and a groove along the dorsal surface of the proximal ischium for the ADD2 origin. Our phylogenetic character tracing across Sauropsida (Supporting Information .nex file) revealed derived musculoskeletal traits for *Postosuchus* (Table 2), including an enlarged cnemial crest on the tibia for the triceps femoris insertions, a rugosity on the craniolateral proximal fibula for the ILFB insertion (ancestral for Suchia), a PIFI1 origin mainly from the ventrolateral ilium, a PIFI2 origin from the “lumbar” vertebrae, centra, and transverse processes (ancestral for Suchia), a “brevis fossa” on the ilium for the lateralized CFB origin, GE, GI, and FL insertions mainly concentrated onto the calcaneal tuber as digit V is reduced, and FHL origins expanded from the caudolateral distal femur onto the lateral cnemial crest of tibia, fossa flexoria, and proximal fibula.

Many archosauriforms, including *Postosuchus*, have a rugose structure projecting ventrally from the dorsal rim of the ilium above the acetabulum. This “supra‐acetabular buttress” (sensu Weinbaum, 2013 = “subvertical rugosity”; Gower & Schoch, 2009) morphology in archosauriforms follows a continuum (Gower, 2000; Nesbitt, 2011: characters 265–267; Weinbaum, 2013: their fig. 17) from a negligible crest (albeit perhaps with an ontogenetic influence) to a prominent ventrally extended crest. This extreme state, with the buttress contributing to the supra‐acetabular crest, and thus, the acetabular articulation, notably occurs in “rauisuchians.” Several studies of archosaur morphology (including Weinbaum, 2013) have interpreted the derived form of the supra‐acetabular buttress to indicate an expanded origin of M. iliofemoralis (IF). Rather, considering that the crest is essentially a ventral extension of the roughened dorsal margin of the ilium, generally regarded as an osteological correlate of the M. iliotibialis (IT2) head's origin, we infer the crest as indicative of a ventrally extended origin of IT2 (Figures 4 and 5; see also Chatterjee, 1985). Furthermore, we infer that the dorsal ilium cranial to the supra‐acetabular buttress remained the IT1 origin (Figures 4 and 5; see also e.g., Galton, 1969; Grillo & Azevedo, 2011a; Piechowski & Tałanda, 2020). This differing interpretation of the IF versus IT origins is analogous to inferences for some ornithischian dinosaurs with an “antitrochanter” (Dilkes, 2000; Galton, 1969; Norman, 1980; Romer, 1923b, 1927) or “supratrochanteric flange” (Maidment & Barrett, 2012). However, the supra‐acetabular buttress should separate muscle origins dorsal and caudal to the acetabulum (e.g., IF; Figures 4 and 5) versus the preacetabular muscle origins (e.g., IT1; PIFI1). This separation also aids interpreting the boundary between the IF muscle origin and the next muscle group, cranial to the IF, as follows.

The apomorphic (slight) cranial expansion of the preacetabular ilium supports the inference of some migration of the M. puboischiofemoralis internus (PIFI1) onto the lateral side of the ilium of *Postosuchus*, cranial to the IF origin and supra‐acetabular buttress (Figures 4 and 5). This PIFI1 origin is convergent with some poposauroids (Schachner et al., 2011) and some dinosaurs (Bishop, Cuff, et al., 2021; Carrano & Hutchinson, 2002; Rowe, 1986). Some of the PIFI1 origin likely remained medially on the pelvis, as is plesiomorphic for archosaurs (Hutchinson, 2001a). However, we judge that much of the PIFI2 origin remained on the ventrolateral surfaces (transverse processes) of the caudalmost dorsal vertebrae, as in Crocodylia, because of the large space ventral to the ventral rim of the preacetabular ilium (Figures 4, 5, 6). That space is only closed off—requiring a shift of the PIFI2 origin from the vertebrae to the lateral ilium—in some theropod dinosaurs, particularly avetheropods, leading to fully lateral PIFI1 and PIFI2 origins in early Avialae (Hutchinson, 2001a, 2002). This inference is contrary to inferences of fully lateral PIFI1 or PIFI2 origins for other archosaurs, including the avemetatarsalian (Figure 1) *Silesaurus* (Piechowski & Tałanda, 2020) and dinosaurs (e.g., some ornithischians; Maidment & Barrett, 2012). It agrees with other reconstructions such as for the early archosauriform *Euparkeria* (Demuth et al., 2022), the “rauisuchian” *Prestosuchus* (Liparini & Schultz, 2013) and the early theropod dinosaur *Staurikosaurus* (Grillo & Azevedo, 2011a).

Certain traits of pelvic limb myology are pertinent in the context of transformations from ancestral traits in other suchians or “rauisuchians” (particularly if *Postosuchus* was digitigrade and/or bipedal) and later transformations associated with the origin of Crocodylomorpha and “sphenosuchians.” Relative to other known “rauisuchians,” *Postosuchus* has a more conspicuous “brevis” shelf or fossa on its postacetabular ilium (Figure 5), associated with a more lateralized M. caudofemoralis brevis (CFB) origin (Hutchinson, 2001a; also seen in some other pseudosuchians including crocodylomorphs; Lecuona et al., 2016; Spiekman et al., 2024). *Postosuchus* has a craniolateral pit on the shaft of the proximal tibia roughly where a deeper, distal part of the long digital flexor muscles (most likely FHL; e.g., Hattori & Tsuihiji, 2021) might have had its origin (Figures 4 and 5; Peyer et al., 2008). Like some “rauisuchians” and related suchians, the medial side of the proximal tibial shaft has a pit (Peyer et al., 2008; also in *Batrachotomus*: Gower & Schoch, 2009; *Prestosuchus* (Figure 1): Roberto‐Da‐Silva et al., 2020; Mastrantonio et al., 2024; possibly in *Rauisuchus* (Figure 1)*:* Lautenschlager & Rauhut, 2015; and the potential poposauroid *Mandasuchus*: Butler et al., 2017) that may correspond to the insertion of the internal parts of the flexor cruris muscles (e.g., FTI1, PIT; Figures 4 and 5).

Plesiomorphically in archosaurs (see Hutchinson, 2001b), the IF insertion was a small tuberosity on the lateral side of the femoral shaft (e.g., in the early archosauriform *Garjainia*: Maidment et al., 2020; in the suchian *Mandasuchus*: Butler et al., 2017; possibly also in other “rauisuchians”: *Batrachotomus*: Gower & Schoch, 2009; *Fasolasuchus* (Figure 1): Bonaparte, 1981; and *Saurosuchus* (Figure 1): Sill, 1974). A derived insertion is more proximally and cranially on the femur (in the potential poposauroid *Smok*; Niedźwiedzki et al., 2011), where a “lesser trochanter” evolved independently in multiple archosaur clades (Hutchinson, 2001b; see also von Baczko et al., 2025). Jalil and Peyer (2007) considered a rugosity on the craniomedial proximal femoral shaft of the “rauisuchian” *Arganasuchus* (see also Dutuit, 1979) to be a “lesser trochanter” (IF insertion), but this position corresponds better to the PIFI1 insertion, which can have scars in archosaurs (Hutchinson, 2001b). We infer a more plesiomorphic IF insertion for *Postosuchus* (Figures 4 and 5; Hutchinson, 2001b), based on the hint of a slight knob on the caudolateral side of the femoral shaft on the TTU‐P 9000 specimen (which may have more of an intermediate character state along the lateral side of the proximal femoral shaft in some taxa, such as in *Mandasuchus*: Butler et al., 2017), the apparent prevalence of this trait in related “rauisuchians” and the absence of evidence for a “lesser trochanter.”

*Postosuchus* lacks many hindlimb features characteristic of “sphenosuchians”—for example, there is no elongate preacetabular crest, strongly rounded and craniomedially facing femoral head with a distinct neck, “pseudointernal trochanter” related to the M. puboischiofemoralis externus insertion, or “lesser trochanter” related to the IF or PIFI2 insertion (Colbert et al., 1952; Walker, 1970; Crush, 1984; Parrish, 1991; Clark & Sues, 2002; Sues et al., 2003; Göhlich et al., 2005; Lecuona et al., 2016; Ruebenstahl et al., 2022; Spiekman, 2023; Spiekman et al., 2024). Some crocodylomorphs and other archosaurs have a groove on the caudal (proximal) side of calcaneal tuber as *Postosuchus* does, correlated with the paths (Figures 4, 5, 6) of the gastrocnemius muscles (GI, GE) and digital flexors (FHL, FDL) (e.g., Lecuona et al., 2016; Schachner et al., 2011, 2020; Spiekman et al., 2024; Walker, 1970). This tendon path might also correlate with the reduction of the calcaneal tendon's attachment (shared by GE and GI) to digit V, which has strongly reduced its phalanges in *Postosuchus* (Chatterjee, 1985; Peyer et al., 2008). Otherwise, *Postosuchus*'s hindlimb morphology either lacks notable distinctions from that of early crocodylomorphs; or, conspicuously, lacks many clear muscle scars; for uncertain taphonomic, functional, ontogenetic, or phylogenetic reasons.

### 3.2 Mass properties of the model and their implications for bipedalism

Figure 7 shows the final whole skeleton and the whole‐body model and its COM. Our model's total body mass was estimated at 396.2 kg. This mass is considerably more (64%) than an estimate of 238.8 kg (25.6% prediction error) from humeral and femoral minimal diaphyseal circumferences (~81 and 147 mm) obtained using equation 1 of Campione and Evans (2012) for quadrupeds; or 193.1 kg (33.8% prediction error) using equation 7 of Campione et al. (2014) for bipeds. It also differs from Chatterjee's (1985) speculation of 250–300 kg body mass.

Our model's body mass estimate also greatly exceeds the estimated 55.41 kg body mass from Henderson and Snively (2004) used in Bishop et al. (2020). Additionally, the COM position in our model (in its default, digitigrade pose with proximal limb joints abducted 15°) is more craniad (0.3857 m from hips here, vs 0.2207 m in the smaller model; see below); whereas the torso is longer (gleno‐acetabular distance = 0.985 m in our model vs 0.62 m) as are the forelimbs and hindlimbs (lengths = 0.49 m and 0.92 m in our model vs 0.353 m and 0.795 m). The specimen used in Henderson and Snively (2004) was not specified, but considering the relative dimensions of our model using TTU‐P 9000, it must have been a markedly smaller specimen (or using previously chimeric reconstructions involving *Shuvosaurus* and *Poposaurus*), such as TTU‐P 9002; consistent with our measurements of scanned bones for that specimen. Similarly to the femur circumference‐based estimates, then, the mathematical slicing‐based estimates of body mass in *Postosuchus* from Henderson and Snively (2004) seem underestimated: using the ratio of femur length versus circumference in specimens TTU‐P 9000 versus TTU‐P 9002 as a scaling factor, cubed to estimate the expected body mass of specimen TTU‐P 9000 from that of TTU‐P 9002, we still only obtain 128.5 kg. Therefore, in the case of *Postosuchus kirkpatricki*, our modelling suggests that alternative methods for estimating its body dimensions (e.g., scaling equations from bone dimensions) might be less plausible, perhaps because of its gracile limbs but robust body.

We also used our whole‐body model to test how suited its COM was for bipedal support. To incorporate a slightly more biologically realistic (but still digitigrade) pose, we adjusted the initial limb pose of the model to −10° flexion of the hip, knee, ankle, wrist, elbow, and 10° extension of the shoulder. The resulting whole body's COM was 0.395 m craniad (and 0.087 m ventrad) to the acetabula, compared with a femur length 0.507 m; that is, about 78% of femur length craniad. Based on these measurements and the logic of Otero et al. (2019), *Postosuchus* might not have been able to sustain bipedalism in the digitigrade pose used, because it might not have been able to place its COM over its feet (the toe tips are about 0.20 m in front of the hips; so ~0.20 m behind the COM). Indeed, it may not have been able to place its COM behind its knees (as per Otero et al., 2019) even if its limbs were more flexed than in our simple scenario (although evidence from the “pillar‐erect” hip joints seems to point toward relatively vertically oriented limbs in *Postosuchus*; e.g., Bonaparte, 1984; Parrish, 1986). Using our plantigrade model, with joints set at their default 0° angles (i.e., fully plantigrade), placed the toe tips 0.33 m craniad to the hips; so only ~0.065 m behind the COM. However, our very basic, quasi‐biomechanical approach involves questionable assumptions such as requiring static equilibrium (necessary for bipedal standing, but not for facultative bipedalism; e.g., Demuth et al., 2023) and perhaps that a biped must have its COM behind its knee joint (see Bishop, Cuff, et al., 2021 for commentary).

Furthermore, the relatively cranially positioned COM we obtained clearly is caused by the robust head and neck (12.1% body mass), and long, thick trunk (40.6% body mass; COM is 40% of glenoacetabular distance from the hips), versus the relatively gracile tail (10.7% body mass total) of our model (Figure 7b). The external contours of the model not only followed the reconstruction in Figure 1a but also relied on numerous scans of the available vertebrae (Figure 2; Table 1). Hence, the COM depends on the reliability of our whole‐body model. All vertebrae except the distal caudals have tall neural spines and thus are dorsoventrally tall, making their segment masses larger than they would otherwise be. But the transverse processes are not proportionately expanded, so in dorsal view, the vertebral column is mediolaterally narrow throughout (Figure 7c; also see Methods and Figure 1a). The trunk segment's boundaries were estimated from the dimensions of the pectoral and pelvic girdles, following an elliptical contour in areas (mediolaterally and ventrally) that were not directly constrained by skeletal geometry in our model; namely ribs and gastralia, for which we lacked scan data. These boundaries in our view are reasonable enough in our model, and because the trunk's COM is so close to the whole‐body COM, any changes of its dimensions should have modest impact on whole‐body COM.

However, the neck as reconstructed is, in particular, mediolaterally wide. Its dorsoventral depth is more directly evident from the tall neural spines of the cervical vertebrae and the deep cranium; and the ventral boundary of the pectoral girdle. Additionally, the proximal tail in our model might be too mediolaterally narrow and not sufficiently tall dorsoventrally. Therefore, we consider the dimensions of the neck and proximal tail to have the greatest potential sources of bias in our COM estimates. To assess the possible impact of these biases, we decreased the mass of the head and neck by 50%, and increased the mass of the proximal tail by 50%, and recomputed the whole‐body COM (Figure 7c). The resulting COM position (in the reference pose) was 0.264 m craniad and 0.0928 m ventrad to the acetabulum, respectively ~68% and 107% of their nominal values. This brought the COM much closer to the hips, although still not over the feet in the reference pose or the more flexed pose. Note that the modelled tail of *Postosuchus* has preserved proximal vertebrae with a ratio of width (across transverse processes) to height (between dorsal center of neural spine to ventral center of centrum) of ~0.53, versus (e.g.) ~0.69 in *Poposaurus* (Yale Peabody Museum [New Haven, CT, USA] specimen YPM 57100), so its tail is relatively gracile (mediolaterally) compared with one bipedal suchian. Table 3 shows results from 3D volumetric models of other early archosaurs and *Crocodylus* (*Euparkeria*, *Gracilisuchus* (Figure 1) and avemetatarsalians *Lagosuchus*, *Heterodontosaurus*, *Plateosaurus*, *Staurikosaurus*, and *Coelophysis*). All these models have larger tails than in *Postosuchus*, even if they are quadrupedal or bipedal (or uncertain).

**TABLE 3:** Relative masses of tails from 3D models of Crocodylia and early archosaurs.

Taxon	Tail mass (kg)	Body mass (kg)	Tail/body mass (%)	References
*Crocodylus johnstoni*	4.98	20.9	23.8	1
*Euparkeria*	0.13	0.90	14.0	2
*Gracilisuchus*	0.232	1.26	18.4	3
*Batrachotomus*	36	190.1	18.9	4
*Postosuchus*	42	396	10.7	here
*Riojasuchus*	6.4	24.56	26.1	5
*Lagosuchus*	0.041	0.134	30.6	6
*Heterodontosaurus*	0.70	4.5	15.5	7
*Plateosaurus*	144	753	19.1	7
*Mussaurus*	349	1418	24.6	8
*Staurikosaurus*	3.7	21.3	17.5	7 (and 9)
*Coelophysis*	3.1	13.1	23.6	10

*Note*: References: 1 = Allen et al. (2009); 2 = Demuth et al. (2023); 3 = Lecuona et al. (2025); 4 = Bishop et al. (2020); 5 = von Baczko et al. (2025); 6 = Otero et al. (2024); 7 = Allen et al. (2013); 8 = Otero et al. (2019); 9 = Grillo and Azevedo (2011b); 10 = Bishop, Cuff, et al. (2021). *Euparkeria*, *Gracilisuchus*, *Batrachotomus*, potentially *Postosuchus*, *Riojasuchus*, and *Mussaurus* all involve substantial reconstruction of missing caudal vertebrae and thus are rough estimates.

Next, we consider how our revised COM data impact classification as a biped or quadruped, using the broad archosauromorph dataset (volumetric models of 80 taxa) from Bishop et al. (2020); mentioned in the Introduction. The latter study used a 3D model that was apparently based on the juvenile TTU‐P 9002 specimen (as per above) in a training dataset for linear discriminant analysis (LDA) and found that *Postosuchus* was “strongly supported as a biped.” They obtained an 84.7% posterior probability of correct classification (i.e., bipedalism) with the study's generally best‐performing “Model 21” (or ~ 98% in their other well‐performing models). We reran their analysis with our amended model's data, instead of the prior (juvenile) model's, which used a different method (mathematical slicing; Henderson & Snively, 2004). The LDA only predicted bipedalism for *Postosuchus* 7 out of 22 times (37.5%), versus quadrupedalism 15 out of 22 times. We did not obtain qualitatively different results from that when using the more caudally positioned COM explained above instead (bipedalism 6/22 times). Thus, this method does not strongly clarify the stance of *Postosuchus*, but as the COM results described above hint, somewhat favors a quadrupedal (or facultatively bipedal) stance.

**FIGURE 8:**
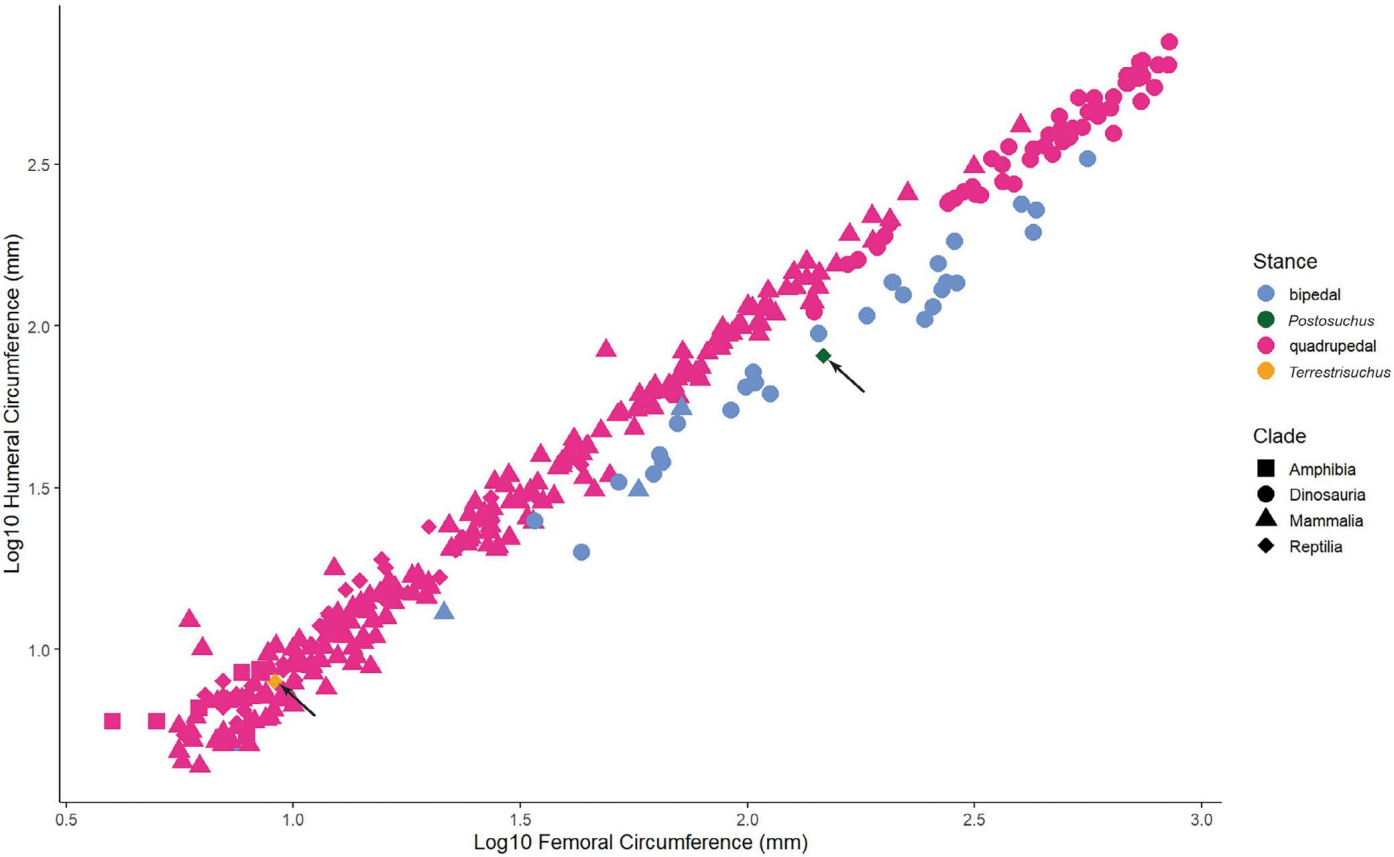
Results from plotting log_10_ humeral versus femoral minimal diaphyseal circumferences for bipeds, quadrupeds, two pseudosuchian archosaurs (arrows), *Terrestrisuchus* (Spiekman et al., 2024), and *Postosuchus* (our data). See Lecuona et al. (2025) for further analyses of other taxa.

As an interesting contrast, we found that *Postosuchus* plotted clearly within the bipedal morphospace from Spiekman et al.'s (2024) database of stylopodial circumferences (Figure 8). The contradiction between these results and those obtained using Bishop et al.'s (2020) analyses implies that either *Postosuchus* has unusually slender humeri or thick femora and actually was quadrupedal, or that its relatively cranially positioned COM in our model (and perhaps its ratio of forelimb vs hindlimb lengths) did not prevent it from moving bipedally. Either way, one of these signals is misleading. Our new results from Bishop et al.'s (2020) method also contradict the results of Pintore et al.'s (2022) 3D geometric morphometrics results, which indicated bipedalism in the adult TTU‐P 9000 specimen that we modelled here. Our results cannot exclude the possibility of facultative bipedalism, or an ontogenetic shift from juvenile quadrupeds to adult bipeds (e.g., Hartman et al., 2022); or perhaps a more modest functional shift in locomotor dynamics.

### 3.3 Assessment of limb joint function and orientation via morphology and ROMs

**FIGURE 9:**
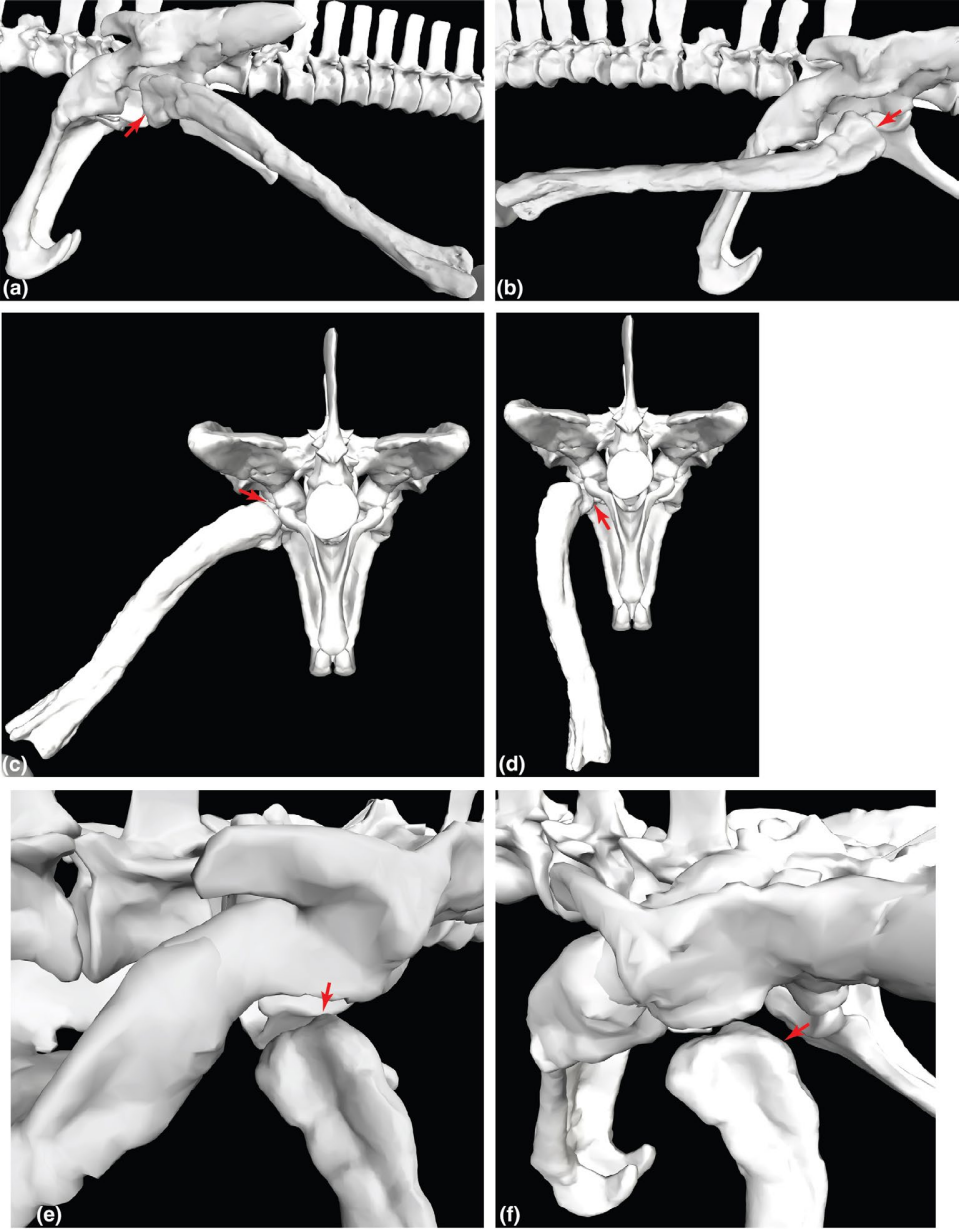
Simple estimates of left hip joint ROMs for *Postosuchus*; and related morphological traits. Maximal and minimal angles for: (a) hip extension (60°; lateral view); (b) hip flexion (−80°; lateral view); (c) hip abduction (50°; caudal view); (d) hip adduction (0°; caudal view); (e) hip external LAR (50°; craniolateral view); and (f) hip internal LAR (−40°; dorsal view). Red arrows indicate articular interactions (contact/disarticulation) used to approximate ROM limits. Not to scale.

As Figure 9 shows, *Postosuchus* has the strongly “pillar‐erect” hip joint morphology characterized by Benton and Clark (1988) and Bonaparte (1984) for some Pseudosuchia such as “rauisuchians” (see Gower, 2000); involving an acetabulum that faces somewhat ventrally, not simply laterally, and that is deemed to favour a more vertically oriented, strongly adducted femur (Chatterjee, 1985). Demuth et al. (2020) argued that some amount of pillar‐erect hip function was ancestral for Eucrocopoda (i.e., *Euparkeria*, Archosauria and closely related taxa; Figure 1), and that remains an accurate description on the basis of a more tightly fitting hip joint than in most earlier Archosauromorpha; but only some suchians such as aetosaurs and “rauisuchians” evolved the most extreme morphology originally described by Benton and Clark (1988) and Bonaparte (1984).

Figure 9 shows the ROMs that we estimated for the hip, with maximal ROM of 140° for flexion/extension, 50° for ab/adduction, and 90° for long‐axis rotation. We placed the hip's 3D joint center (Figure 3) in the 3D centroid of the acetabulum, with a relatively large space around the femoral head (representing articular soft tissues; see Tsai & Holliday, 2014; Demuth et al., 2020), so ROMs were greater than if we had positioned it more medially. To the degree that these ROMs are reliable for higher level inferences, they suggest relatively wide mobility of the hip, with maximal retraction bringing the femur's long axis roughly parallel with the ischium's (Figure 9a) or maximal protraction having the femur nearly parallel with the iliac craniocaudal axis (Figure 9b). As expected, hip abduction and adduction are, respectively, strongly limited by the supra‐acetabular crest (Figure 9c) and the ventral acetabular rim (Figure 9d). Internal/external rotation limits are more subjective, but seem limited by the antitrochanter (Figure 9e,f). Tsai and Holliday (2014) showed that a perforated acetabulum, as in *Postosuchus*, indicates the presence of pubofemoral and ischiofemoral intracapsular ligaments, rather than an erect hindlimb posture. Future reconstructions of these ligaments could help constrain estimates of hip joint mobility in *Postosuchus* (e.g., see Demuth et al., 2025).

**FIGURE 10:**
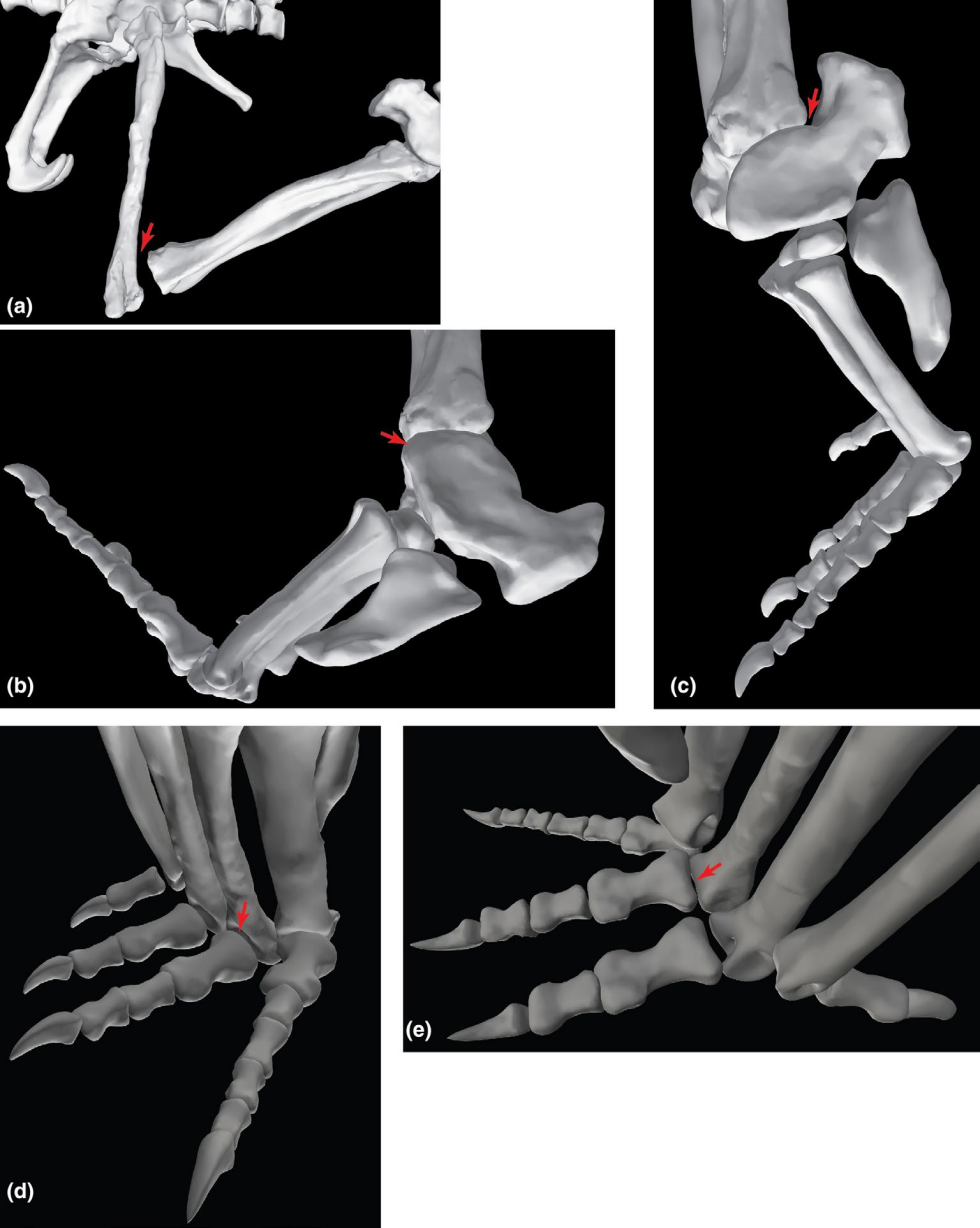
Simple estimates of left lower hindlimb joint ROMs for *Postosuchus*; and related morphological traits. Maximal and minimal angles for: (a) knee flexion (−110° in lateral view; extension is 0°); (b) ankle flexion (−50°; lateral view); (c) ankle extension (30°; lateral view); (d) third metatarsophalangeal joint extension (dorsiflexion −30°; craniolateral view); and (e) third metatarsophalangeal joint flexion (plantarflexion 160°; caudomedial view). Red arrows indicate articular interactions (contact/disarticulation) used to approximate ROM limits. Not to scale.

The distal end of the femur and proximal tibia have several traits that hint toward more parasagittal knee function. The femur has a deep intercondylar groove and distinct, caudally extensive condyles, and low torsion overall (Parrish, 1986; Pintore et al., 2022). The lateral condyle projects further distally than the medial condyle, which would favor adduction of the tibia and fibula (via a medial incline to the knee axis), thereby facilitating more medial foot placement in a more erect hindlimb posture. There is a prominent, caudally facing fibular condyle (crista tibiofibularis) with a fossa separating it from the lateral condyle on the distal end (Parrish, 1986; Pintore et al., 2022). Figure 10a shows the flexion ROM (maximum −110°) we approximated for the knee, limited by articulation of the tibial (and fibular) plateau and distal condyles (an extension angle of 0° as in Figure 3 was considered maximal extension; see Lecuona et al. (2025) for further justification). That flexion ROM is difficult to objectively estimate given the space provided by cartilage.

The distal tibia and proximal tarsals add further evidence for more parasagittal limb (here, ankle) joint function in *Postosuchus*; such as the tibia positioned vertically relative to the astragalus, with its shaft perpendicular to the mediolateral (flexion/extension) axis of the ankle, and pronounced articular surfaces for the proximal tarsals on the distal fibula, among other traits (see Parrish, 1986). Our estimated joint axes (Figure 3) are less oblique to each other than in *Euparkeria* (Demuth et al., 2020), again more consistent with an erect hindlimb posture and relatively parasagittal gait. Parrish (1986) noted the general morphofunctional similarity of *Postosuchus* to other suchian archosaurs with more parasagittal hindlimb function, yet that the more medial orientation of the calcaneal tuber in *Postosuchus* might have facilitated a stronger focus on ankle flexion/extension. In our digitigrade model, the calcaneal tuber is at a ~ 30° angle to the mediolateral axis of the astragalocalcaneal joint (itself slightly oblique, at ~10° to the whole‐body mediolateral axis), supporting Parrish's (1986) point. Figure 10 shows the ROMs (total 85°) we estimated for the ankle, considered in a comparative context below, with Figure 10b approximating maximal flexion when the distal tarsals contact the astragalus and the calcaneum loses proper articulation with the distal fibula. As in many other archosaurs with large calcaneal tubers (e.g., the ornithosuchid (Figure 1) *Riojasuchus* in von Baczko et al., 2025), the caudal projection of the tuber would have limited ankle extension, because it came to contact the caudal side of the fibula as the ankle approached hyperextension and, proper fibular‐calcaneal articulation again is lost (Figure 10c).

Finally, the third metatarsophalangeal joint's total of 190° ROM is, as typical for archosaurs and other tetrapods, limited in dorsiflexion by the hyperextension surface on the metatarsal condyle (Figure 10d) and in plantarflexion by the proximally expanded plantar articular surface of the condyle (Figure 10e).

### 3.4 Plantigrade versus digitigrade *Postosuchus*?

**FIGURE 11:**
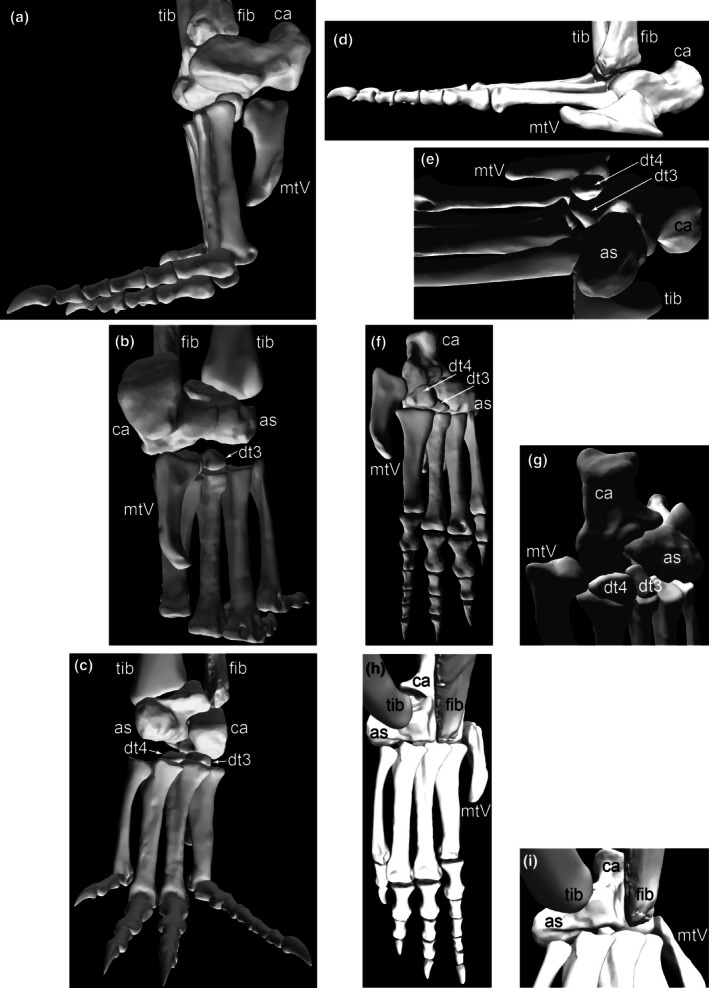
Comparison of left digitigrade and plantigrade pes articulations modelled for *Postosuchus*. Digitigrade pes in lateral (a) caudal/ventral (b) and cranial/dorsal (c) views; plantigrade pes in lateral (d) caudal/ventral (e–g) and cranial/dorsal (h, i) views. as, astragalus; ca, calcaneum; dt3, distal tarsal III; dt4, distal tarsal IV; fib, fibula; mtV, metatarsal V; tib, tibia. Not to scale.

We used different models of the pes of *Postosuchus* to explore our study's question about which pes orientation was more plausible. Note that our model used metatarsal III (and other elements of the pes) from the TTU‐P 9002 specimen, whereas metatarsal I, II, IV, and V came from NCSM 13731 (*Postosuchus allisonae*), so we interpreted our results for articulations carefully. Our original digitigrade model versus the modified plantigrade model had identical positions for all skeletal elements except the calcaneum, distal tarsals, metatarsals, and (rearticulated) phalanges (Figure 11). All models had the astragalus as part of the “crus” segment (with tibia and fibula), whereas all other tarsal and metatarsal bones were modelled in one “pes” segment; no intra‐segment motions were possible in any models. However, digit I was articulated as part of the pedal digits in the plantigrade *Postosuchus* model rather than immobile on the pes as it was in the digitigrade model, so digit I's metatarsophalangeal joint moved with the other digits (i.e., focused on digit III). The bone we identified as distal tarsal IV was only a little larger than distal tarsal III (both came from TTU‐P 9002). It was not large enough to articulate “with the fifth, fourth and third metatarsals, and the calcaneum” (p. 426 in Chatterjee, 1985). This relatively smaller size seems unusual for archosaurs, and distal tarsal IV's articular surfaces were difficult to interpret but we placed it in the usual position atop the proximal end of metatarsal IV. It is possible that this bone is from a different specimen or taxon.

The digitigrade *Postosuchus* model had metatarsal V on the caudolateral side of the pes as in some other early archosaurs (Sereno, 1991)—for example, *Poposaurus* (Figure S2), *Effigia* (Nesbitt, 2011), the “sphenosuchian” *Hallopus* (Walker, 1970), *Lagosuchus* (Sereno & Arcucci, 1994), close to articulating with distal tarsal IV's caudal surface, and its distal “hooked” end pointed toward the caudal side of metatarsal IV (Figure 11a,b). The left hindlimb of the YPM 57100 *Poposaurus* specimen has a similar articulation preserved (e.g., Schachner et al., 2020: their fig. 2) and modelled (Bates & Schachner, 2012; Farlow et al., 2014). Although metatarsal V's proximal articular surface also approached contacting the flat distal fossa of the calcaneum (Figure 11a,b), the absence of calcaneal–metatarsal motion in our *Postosuchus* model did not allow this articulation to move and thus make contact. Distal tarsal bones III and IV were placed in fairly conventional locations over their articular surfaces on the corresponding proximal metatarsals III and IV; and thereby having their proximal articulations mainly with the astragalus and calcaneum, respectively (Figure 11b,c). The calcaneum was in close articulation with the astragalus (medially) and distal end of the fibula (proximally).

The plantigrade *Postosuchus* model was different in that metatarsal V was articulated on the lateral side of the tarsus, close to contacting the lateral side of the distal calcaneum and the lateral sides of distal tarsal IV and the proximal end of metatarsal IV (Figure 11d–g). Additionally metatarsal V's distal tip curved toward the lateral shaft of metatarsal IV (Figure 11d–f,h). Generally, in this plantigrade model, metatarsal V's position represented the plesiomorphic state for archosaurs (e.g., Nesbitt, 2011; Sereno, 1991). An unusual outcome of these articulations was that the reference pose of the model had the pes strongly “toed‐out” (abducted) relative to the parasagittal plane; by ~22°; unlike the digitigrade pose (Figure S3). Allowing at least one extra DOF at the ankle (abduction/adduction) could remove this orientation, which is purely an outcome of modelling conventions and not indicative of a most likely biological pose of the pes. Our ROM analysis of this *Postosuchus* model allowed ankle (dorsi)flexion from −35° (when metatarsals I and IV contacted the proximal tarsals) to (plantarflexion) extension of 50° (when the calcaneal tuber contacted the caudal side of the distal fibula). This ROM (total of 85°) is similar to the ankle ROM in the digitigrade pose (from −50° to 30°; total ROM of 80°), which is easily within the plausible range of error in manual ROM analysis with a somewhat simple model of the ankle. Hence, our ROM analysis does not favor either pes pose in *Postosuchus*.

Overall, the pes structure of *Postosuchus* is similar to that of other erect‐limbed suchians (e.g., ornithosuchids, aetosaurs (Figure 1), “sphenosuchians,” “rauisuchians”; Krebs, 1965; Walker, 1970; von Baczko et al., 2019; Spiekman, 2023; Spiekman et al., 2024), including some bipeds such *Poposaurus* (Schachner et al., 2020). Peyer et al. (2008) interpreted the *P. alisonae* pes as plantigrade without further commentary. Metatarsal V's plesiomorphic “hooked” morphology is reduced, but the bone remains robust as in many archosauriforms (e.g., Nesbitt, 2011). The metatarsals are somewhat bunched and metatarsals I–IV are approximately parallel (Figure 11), similar to *Poposaurus* (Figure S2; Farlow et al., 2014). By Nesbitt's (2011, p. 174) definition of a “compact metatarsus as metatarsal II–IV contacting each other for at least the proximal half of the elements,” *Postosuchus* does not have a compact metatarsus (Figure 11). Consequently, via the criteria of Turner and Gatesy (2021), this would favor a plantigrade pes. Chatterjee (1985) reconstructed *Postosuchus* as digitigrade primarily on the basis of its mesaxonic pes (i.e., dominated by digits II–IV; typically in tandem with a compact metatarsus), citing similarities with theropod dinosaurs. This is an interesting correlate of digitigrady—also evident in many “sphenosuchians” (e.g., Walker, 1970) and some other archosaurs, especially ornithodirans—but still does not demonstrate digitigrady. In contrast, articulated pedes of some “rauisuchians” such as *Prestosuchus* (Desojo et al., 2020; Roberto‐Da‐Silva et al., 2020) and *Decuriasuchus* (França et al., 2011) suggest a plantigrade pes (Bonaparte, 1984; Nesbitt et al., 2013), and perhaps thereby circumstantial evidence for plantigrady in *Postosuchus*—and particularly, the articulated pes of *P. alisonae* intimates plantigrady (Peyer et al., 2008).

Key reasoning for the (at least facultatively) digitigrade hypothesis for *Poposaurus* (see Figure S2), which also can be applied to *Postosuchus*, is that (Gauthier et al., 2011; Schachner et al., 2020): (1) it had an expanded calcaneal tuber with a lateral insertion for “peroneus longus” (=FL here) and a groove for the GI and GE tendons; (2) its tight articulations for tibia, fibula, and proximal tarsals might prohibit non‐parasagittal motions; (3) its wide ROM for the ankle and metatarsophalangeal joints allowed plantigrade and digitigrade poses; (4) a plantigrade pose would excessively compress the calcaneal tendon; (5) plantigrady involves contact of the reduced metatarsal V with the ground but metatarsal V seems non‐weight‐bearing. We do not consider this evidence compelling for *Postosuchus*. On points 1, 3, 4, and 5, these traits (or lack thereof) are similarly (qualitatively) evident in plantigrade as well as digitigrade archosaurs; it is not clear that they are exclusively indicative of digitigrady. On point 2, Turner and Gatesy (2023) noted that *Postosuchus* fit their morphological criteria for a “complex 2” tarsus (shared by possibly digitigrade taxa such as *Poposaurus*, “sphenosuchians” and ornithodirans [avemetatarsalians]). “Complex 2” would involve retention of non‐trivial amounts of abduction/adduction and long‐axis rotation across the crurotarsal region, with one main medial joint (astragalus‐metatarsal I) versus perhaps three main lateral joints (astragalocalcaneal, mesotarsal [~calcaneal‐distal tarsals] and tarsometatarsal [calcaneum/distal tarsal IV‐metatarsal V]). Our simple model of ankle joint ROM excluded other tarsal/metatarsal motions that “Complex 2” of Turner and Gatesy (2023) would involve if modelled more realistically. Following this inference and our qualitative analysis above, there remains potential 3D mobility in the distal tibial/fibular and tarsal articulations of *Postosuchus*. Our ROM analysis supports point 3 but this does not conclusively indicate digitigrady. No biomechanical analysis or otherwise demonstrates that a plantigrade pes in *Postosuchus* would risk damage to the calcaneal tendon (point 4), and we consider this risk unlikely given the soft tissues surely present around the heels. A reduced metatarsal V is evident in extant, plantigrade Crocodylia (as well as potentially plantigrade proterochampsians; e.g., Nesbitt, 2011, character 399), and metatarsal V in *Postosuchus* remains remarkably robust (Figure 11), so point 5 is particularly unconvincing for *Postosuchus*. However, the morphometric analysis of pedal elements by Farlow et al. (2014) found that *Poposaurus* had dimensions such as a short metatarsal I and a non‐gracile digit IV with short phalanges that made it most similar to digitigrade early dinosaurs. Farlow et al. (2022) found, with a more expanded analysis, that *Postosuchus* overall had a relatively shorter pes than *Poposaurus*, but still had similarities to the pedal dimensions of early, digitigrade dinosaurs.

**FIGURE 12:**
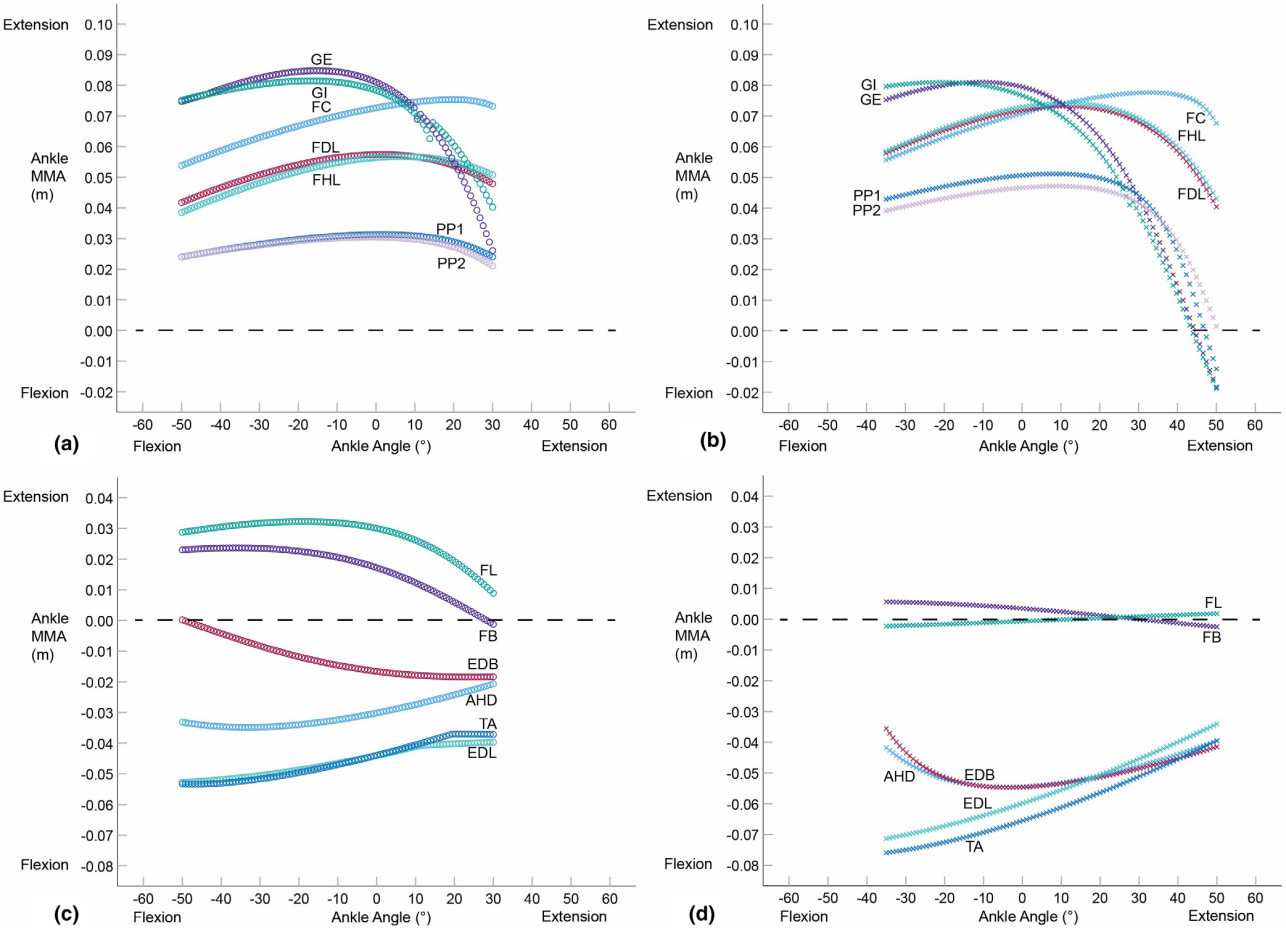
Ankle muscle moment arms (MMAs) for *Postosuchus*, testing for effects of pes orientation. Dashed horizontal line clarifies MMA of zero. (a) Extensors in digitigrade pose; (b) Extensors in plantigrade pose; (c) Flexors in digitigrade pose; (d) Flexors in plantigrade pose. See Table 2 for acronyms.

Our original digitigrade musculoskeletal model versus the plantigrade version for *Postosuchus* involved somewhat different muscle paths because of repositioned bones and horizontal pes orientation. We tested for differences in ankle flexion/extension muscle moment arms (MMAs). Notably, the average MMAs (across the full joint ROMs) of the digitigrade versus plantigrade models (Figure 12) were larger for the FB (~7.4 times) and FL (~110 times, because the FL had almost a zero MMA in the plantigrade pose), with the GE and GI MMAs also slightly larger (1.24 and 1.29 times). However, the nine other pes muscles present in both models (AHD, EDB, EDL, FC, FDL, FHL, PP1, PP2, and TA) uniformly had smaller MMAs (average 0.71 times; median 0.76 times) for the digitigrade model. The four muscles acting around the MTP joint in both models (see Supporting Information) had similar MMAs across the MTP's ROM (average ~ 0.96 ratio digitigrade/plantigrade). The EHL and FHB had MMAs around the MTP joint in the plantigrade model too (averages of −0.023 m and 0.033 m, respectively; similar to other pes muscle MMAs), whereas they did not in the digitigrade model because digit I was modelled out of contact with the ground, immobile as part of the pes rather than digits. Key ankle extensors GE and GI reduced their MMAs steeply with strong ankle extension; more steeply in the plantigrade pose (Figure 12b); and their weaker synergists PP1 and PP2 had a similar pattern.

Thus while the digitigrade *Postosuchus* model's morphology gave the model some advantages for greater MMAs of four key ankle extensors (FB, FL, GE, GI), it had trade‐offs for smaller MMAs of the others (Figure 12), and lacked any effective MMAs for hallucal muscles because of an assumed lack of support against the substrate for the MTP joint from those intrinsic muscles. We revisit this issue below when estimating muscle sizes, because effects of pes orientation on MMAs might manifest differently in the capacities of those muscles to produce moments around joints, as some muscles will be larger or smaller than others (across both models). Regardless, a clear morphofunctional implication of the digitigrade pes (as modelled) is that the repositioned MT V turns FL and FB into ankle extensors more than the ankle abductors they were in the plantigrade model. This ankle extension capacity (with increases in GE and GI leverage too) would aid in antigravity support during digitigrady, although some other muscles may have had decreased leverage.

### 3.5 Quadrupedal versus bipedal *Postosuchus*?

As we reviewed in the Introduction, there is no consensus on whether (adult) *Postosuchus* was habitually quadrupedal (e.g., Brusatte et al., 2009, 2010; Butler et al., 2011; Long & Murry, 1995; Nesbitt et al., 2013; Parrish, 1986), facultatively bipedal (e.g., Chatterjee, 1985; seemingly Kubo & Kubo, 2012), or fully bipedal (e.g., Bishop et al., 2020; Grinham et al., 2019; Hartman et al., 2022; Pintore et al., 2022; Weinbaum, 2013). Gauthier et al. (2011) cited extreme forelimb reduction, long hindlimbs, five sacral vertebrae, and loss of dermal armor as indicative of bipedalism in *Poposaurus* and theropod dinosaurs. Whereas short forelimbs and long hindlimbs are correlates of bipedalism, some bipedal archosaurs (e.g., *Lagosuchus*; early theropods such as herrerasaurids) lacked five sacral vertebrae, and it is not clear why reduced dermal armour indicates *bipedalism* (dermal armour was also “re‐evolved” in the thyreophoran *Scutellosaurus*, which plausibly was bipedal; e.g., Anderson et al., 2023). These features do not conclusively identify *Postosuchus* as bipedal. Most traits cited as indicative of bipedalism are only correlative (e.g., forelimb proportions or femur shape; Weinbaum, 2013; Bishop et al., 2020; Pintore et al., 2022) rather than mechanistically causative. While there is a trend in archosaurs for a digitigrade pes to evolve with bipedalism (Kubo & Kubo, 2012), classification of *Postosuchus* as digitigrade would not mandate its identification as bipedal.

A key issue remains whether the manus of *Postosuchus* was able to sustain support and locomotion, which the tightly bound manus suggests (Hutson & Hutson, 2015; Peyer et al., 2008), with the corollary of whether an ability to act in support and motion indicates obligatory quadrupedalism. This is reminiscent of the controversy over quadrupedalism versus bipedalism in ornithopod dinosaurs, some of which have an analogous manus structure (Maidment & Barrett, 2012; Moreno et al., 2007; Norman, 1980). If the manus of *Postosuchus* could support locomotion, that still might simply be consistent with obligate bipedalism (e.g., the manus was only used during sitting) or facultative bipedalism (during slower movement, as several studies cited above have suggested). Notably, presumed bipedal suchians such as *Poposaurus* lack the interlocking metacarpals and reduced phalanges evident in *Postosuchus*, or even lack ossified carpals. This absence slightly weakens the inference of habitual bipedalism in *Postosuchus*, although the “hooflike” manual unguals of *Poposaurus* (Schachner et al., 2020) could be viewed as hinting at some quadrupedal function; as could the lack of carpal ossification, which also occurs in hadrosaurids and some other quadrupedal dinosaurs (Maidment et al., 2012). Unfortunately, comparative material for the manus of other “rauisuchians” is scarce. The manus of *Ticinosuchus* is more plesiomorphic, consistent with quadrupedalism (Krebs, 1965).

Independent lines of evidence regarding bipedalism would be valuable. Our analysis of COM position (Figure 7a,c) favors some quadrupedal abilities but does not falsify habitual or facultative bipedalism. The most valuable evidence could be predictive simulation with a biomechanical musculoskeletal model (e.g., Anderson et al., 2023; Bishop, Falisse, et al., 2021; Polet & Hutchinson, 2022; Sellers et al., 2009), and our model presented here forms the basis for such a procedure.

**FIGURE 13:**
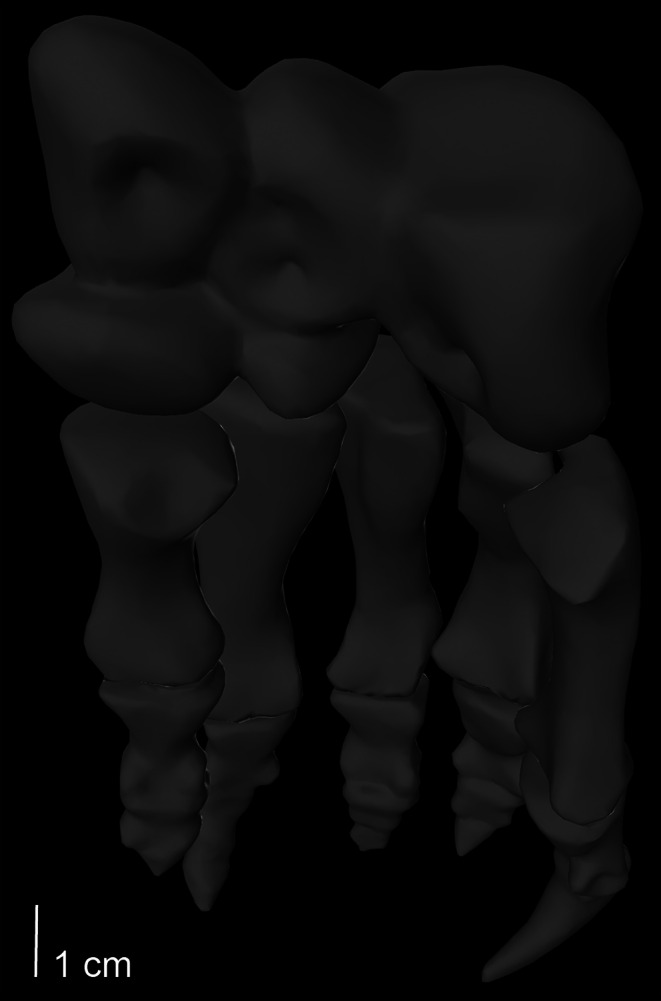
Left manus (NCSM 13731) from our model; in palmar view; showing the “metacarpograde” morphology and the arched structure of the metacarpals, which brings metacarpals I and V palmar to the others.

There are no fossil trackways conclusively attributable to *Postosuchus*. Many trackways have been suggested as being made by “rauisuchians” and some even attributed to a quadrupedal *Postosuchus* (e.g., *Brachychirotherium*; Klein & Lucas, 2010; Hminna et al., 2013; Apesteguía et al., 2021; Klein & Heckert, 2023), but the trackmaker identification as *Postosuchus* depends on what its diagnostic traits are. Indeed, Klein and Heckert (2023) note that *Pseudotetrasauropus* trackways might be from *Postosuchus* if it was bipedal. The small, semi‐pronated “metacarpograde” manus of *Postosuchus* (Figure 13; Hutson & Hutson, 2015) with reduced, blunt phalanges on digits III and IV (combined with a trenchant ungual on digit I) might be one suite of diagnostic traits if quadrupedal trackways exist (see Polet & Hutchinson, 2022), and *P. alisonae* uniquely seems to have six phalanges on pes digit IV (Peyer et al., 2008). Tracks with all of these traits would indicate at least some quadrupedalism in *Postosuchus*. Yet the pes lacks major apomorphies distinguishing *Postosuchus* from other “rauisuchians,” so identifying bipedalism is problematic. However, see Farlow et al. (2022) for a morphometric analysis of archosaur pedes that could help identify *Postosuchus*‐like tracks.

**FIGURE 14:**
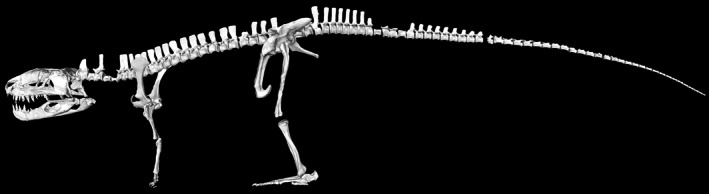
One conceivable quadrupedal, plantigrade static pose for *Postosuchus*. Joints set at: −15° (downward) pelvis pitch, −20° hip flexion, 15° hip abduction, −20° knee flexion, −15° ankle (dorsi)flexion, 10° proximal tail dorsiflexion, 10° neck dorsiflexion, −5° shoulder flexion, 15° shoulder abduction, −15° elbow flexion, and 90° metacarpophalangeal dorsiflexion; all other joints at 0°. Not to scale.

As an initial exploration of potential quadrupedalism in *Postosuchus*, we attempted to pose our model using criteria to bound potential joint orientations within estimated ROMs (forelimbs: Figures S4 and S5) and evidence from joint morphology (e.g., erect, adducted limbs; the vertical manus and pillar‐erect hip). Figure 14 shows one of many possibilities and the joint angles input. Some modest downward pelvic pitch is particularly important to lower the forelimbs to the ground, and the intervertebral articulations appear to have allowed this slight pitch. A plantigrade pes is far more consistent with quadrupedalism than a digitigrade one would be, because the added functional length of a digitigrade pes (0.158 m between JCSs of the ankle and MTP; ~17% of hip height in the plantigrade model) would require quite flexed hindlimbs; inconsistent with a pillar‐erect hip; in order to allow the short forelimbs to contact the ground even in a columnar orientation. Our simple analysis merely suggests that joint morphology seems to allow quadrupedal poses. Whether or not manus muscles (and bones), in particular, could handle playing a supportive role is not yet answerable but a biomechanical model such as ours could address this with further analyses.

In summary, multiple lines of available evidence about plantigrady/digitigrady and quadrupedalism/bipedalism point toward different conclusions for *Postosuchus*. This ambiguity prevents tests of broader ideas about locomotor evolution such as how strongly bipedalism, digitigrady, and “cursoriality” were linked in archosaurs (Gauthier et al., 2011; Kubo & Kubo, 2012) or how “pillar‐erect” hips and bipedalism (and related apomorphies such as hindlimb muscles specialized for parasagittal actions) were linked in pseudosuchians (Bates & Schachner, 2012). Better evidence for *Postosuchus*, and further analyses of archosauriforms such as *Euparkeria* (e.g., Demuth et al., 2020, 2023), proterochampsids and phytosaurs, and pseudosuchians such as aetosaurs, erpetosuchids, other “rauisuchians,” poposauroids, and crocodylomorphs, as well as early‐diverging avemetatarsalians such as aphanosaurs and early‐diverging pterosauromorphs, is needed to provide robust conclusions about fundamental patterns in archosaur locomotor evolution.

### 3.6 Hindlimb muscle mass estimation and comparisons

Table 4 shows our calculations of hindlimb muscle masses reconstructed for *Postosuchus* (Figure 6 **)** in a digitigrade pose, and comparative data from extant Crocodylia; a 278 kg adult Nile crocodile (*Crocodylus niloticus*; from Allen et al., 2014). Note that our reconstruction would encompass tendons (where present), not purely striated muscle bellies, so the muscle (belly) masses are overestimated mainly for distal hindlimb muscles, yet presumably not greatly so because the corresponding bellies are much larger than the tendons in extant Crocodylia (Allen et al., 2014). Indeed, the tendons are only an average of 7.4% of muscle‐tendon unit mass (including only muscles with tendons) in our Nile crocodile used for comparisons; peaking at 36% for the FL tendon (Table 4). Notably, the CFL tendon's mass was 0.039 kg; small relative to the muscle belly (2.35 kg) but about five times larger than the average tendon (0.0078 kg). Our dataset lacked data for the EHL muscle of both taxa and the AHD, FC, and FHB for the crocodile.

**TABLE 4:** Comparative muscle‐tendon unit (MTU) data for a 278 kg Nile crocodile (“Croc”) versus the digitigrade *Postosuchus* model used for volumetric muscle modelling; for one pelvic appendage.

Muscle	Croc muscle mass (kg)	Croc MTU length (m)	Croc tendon mass (kg)	Croc tendon/muscle %M_b_	Croc MTU mass (kg)	*Postosuchus* MTU length (m)	*Postosuchus* MTU mass (kg)	Ratio MTU length
ADD1	0.27	0.031	0.000		0.27	0.36	0.71	23
ADD2	0.050	0.026	0.000		0.050	0.37	0.56	22
AHD						0.15	0.042	
AMB1	0.14	0.017	0.027	20	0.16			
AMB2	0.031	0.017	0.00080	2.6	0.032			
**AMB1+2**	**0.17**				**0.17**	**0.55**	**1.1**	64
CFB	0.067	0.011	0.0022	3.3	0.069	0.27	0.76	69
CFL	2.3	0.049	0.039	1.7	2.3	0.87	5.9	120
EDB	0.088	0.0070	0.0014	1.6	0.089	0.30	0.21	30
EDL	0.10	0.023	0.0020	2.0	0.10	0.44	0.50	22
FC						0.059	0.050	
FB	0.015	0.016	0.0010	6.6	0.016	0.23	0.18	11
FL	0.039	0.017	0.014	36	0.053	0.37	0.37	22
FDB	0.14	0.011	0.022	15	0.17	0.27	0.13	12
FHB						0.20	0.035	
FDL	0.11	0.031	0.016	14	0.12	0.73	0.84	27
FHL	0.025	0.018	0.0044	18	0.029	0.89	0.26	15
FMTE	0.091	0.018	0.000		0.091	0.25	0.66	36
FMTI	0.415	0.018	0.030	7.3	0.45	0.24	1.5	87
FTE	0.30	0.041	0.014	4.7	0.31	0.70	3.0	74
FTI1	0.045	0.025	0.000		0.045	0.51	0.51	20
FTI2	0.17	0.031	0.0032	1.9	0.17			
**FTI1+2**	**0.22**	**0.056**	**0.0032**		**0.22**		**0.51**	
FTI3	0.093	0.023	0.0018	1.9	0.094	0.58	0.60	26
FTI4	0.025	0.027	0.0034	13	0.029			
**FTI3+4**	**0.12**	**0.050**	**0.0052**		**0.12**		**0.60**	
GE	0.33	0.017	0.040	12	0.367	0.56	1.5	89
GI	0.073	0.020	0.0046	6.3	0.077	0.42	0.94	47
IC/PP1	0.13	0.016	0.0060	4.7	0.135	0.35	0.42	27
IF	0.099	0.019	0.000		0.099	0.32	0.51	28
ILFB	0.087	0.024	0.0030	3.4	0.090	0.78	1.3	55
ISTR	0.058	0.018	0.0048	8.3	0.063	0.21	0.24	13
IT1	0.031	0.017	0.0020	6.4	0.033	0.71	1.2	67
IT2	0.25	0.018	0.027	11	0.27	0.72	1.6	87
IT3	0.068	0.030	0.0028	4.1	0.071	0.74	2.2	72
PIFE1	0.045	0.012	0.0010	2.2	0.046	0.39	1.0	88
PIFE2	0.034	0.013	0.0024	7.1	0.036	0.36	1.3	103
PIFE3	0.096	0.013	0.0020	2.1	0.098	0.14	0.18	14
PIFI1	0.18	0.026	0.000		0.18	0.18	0.47	18
PIFI2	0.52	0.025	0.012	2.3	0.53	0.31	1.2	48
PIT	0.13	0.029	0.0040	3.1	0.13	0.58	0.54	19
PP/PP2	0.024	0.007	0.000		0.024	0.26	0.22	32
TA	0.097	0.022	0.000		0.097	0.57	0.67	31
**Total**	**6.8**	**0.023**	**0.27**	**7.4**	**7.1**	**0.43**	**34**	**45**
		**Average**	**0.0078**					

*Note*: Raw *Crocodylus niloticus* muscle architecture data are from dissection (Allen et al., 2014). Tendon versus muscle belly (“muscle”) mass are compared as percentages of body mass (M_b_). Finally, MTU lengths for *Postosuchus* (from OpenSim model) versus. the crocodile are listed. Our model of *Postosuchus* was reconstructed to lack differentiated AMB1+2, FTI1+2, and FTI3+4 so the rows with bold font indicate comparative data for those muscle pairs, each summed in *Crocodylus*. Table 5 provides further comparisons of these data.

Some differences between the MTU masses of the two taxa might arise from different MTU lengths (origin to insertion), and MTU lengths are biomechanically important because greater lengths give greater capacity for length changes and thereby joint rotations. The MTU lengths were measured differently: in *Crocodylus*, muscle and tendon were removed in dissection and measured linearly when flat and straight; in *Postosuchus*, from plotting MTU lengths in the reference pose in OpenSim (so most paths had some curvature). This will introduce some error, but the morphological differences noted above should still produce identifiably stark differences in MTU lengths. Mean ratios of MTU lengths (Table 4) for *Postosuchus* versus *Crocodylus* were very large at 45. Most notably, the CFL was 120 times longer than in *Crocodylus*, followed by 103 times for PIFE2, 89 for GE, 88 for PIFE1, and 87 for FMTI. The smallest ratios were 11 for FB, 12 for FDB, 13 for ISTR, and 14 for PIFE3. All these are fairly short MTUs in both taxa.

Compared with the crocodile (Table 5), our *Postosuchus* model's muscles (as percentages of body mass per hindlimb) were relatively larger overall, by about 3.2 times total (~2.7% vs. 8.6% body mass). This is not surprising if *Postosuchus* was bipedal, with long hindlimbs and thus relatively more body mass dedicated to hindlimb muscles; and its expansive pelvis in comparison with Crocodylia suggests large thigh muscles. However, it is informative to investigate how specific muscles and groups of muscles compare (Table 5). The muscles that were relatively the largest in *Postosuchus* versus *Crocodylus* (as percentages of body mass, 10+ times larger) were (in order from largest) PIFE2, IT1, IT3, PIFE1, and ILFB. These differences persisted if MTU masses were compared as percentages of total hindlimb muscle mass (3+ times larger). The long ilia, pubes, and ischia may explain this pattern because all of these muscles are proximal thigh/pelvic muscles. Only the FDB was relatively larger in the crocodile (ratio 0.54), which may occur because of the more gracile pes in *Postosuchus* (EDB was also less large than other muscles, at a ratio of 1.7).

**TABLE 5:** Comparative muscle‐tendon unit (MTU) data for a 278 kg Nile crocodile (“Croc”) versus the digitigrade *Postosuchus* model used for volumetric muscle modelling; derived from Table 4 data; for one pelvic appendage.

Muscle	MTU %M_b_ Croc	MTU %M_b_ *Postosuchus*	Ratio %M_b_	%MTU mass Croc	%MTU mass *Postosuchus*	Ratio %MTU
ADD1	0.096	0.180	1.9	0.038	0.021	0.55
ADD2	0.018	0.140	7.9	0.0070	0.016	2.3
AHD		0.011			0.0012	
AMB1	0.059			0.023		
AMB2	0.012			0.0045		
**AMB1+2**	**0.060**	**0.273**	**4.5**	**0.024**	**0.032**	**1.3**
CFB	0.025	0.192	7.8	0.010	0.022	2.3
CFL	0.86	1.5	1.7	0.34	0.17	0.51
EDB	0.032	0.054	1.7	0.013	0.0063	0.50
EDL	0.037	0.125	3.4	0.015	0.015	1.0
FC		0.013			0.001	
FB	0.0058	0.046	7.8	0.0023	0.0053	2.3
FL	0.019	0.093	4.9	0.0075	0.011	1.5
FDB	0.059	0.032	0.54	0.023	0.0038	0.16
FHB		0.0088			0.0010	
FDL	0.045	0.212	4.7	0.018	0.025	1.4
FHL	0.011	0.067	6.4	0.0041	0.0078	1.9
FMTE	0.033	0.17	5.0	0.013	0.019	1.5
FMTI	0.16	0.38	2.4	0.063	0.045	0.71
FTE	0.11	0.77	6.8	0.044	0.090	2.0
FTI1	0.016	0.13	8.0	0.0064	0.015	2.4
FTI2	0.063			0.025		
**FTI1+2**	**0.079**	**0.13**	**1.6**	**0.031**	**0.015**	**0.49**
FTI3	0.034	0.151	4.4	0.013	0.018	1.3
FTI4	0.010			0.0041		
**FTI3+4**	**0.044**	**0.151**	**3.4**	**0.017**	**0.018**	**1.0**
GE	0.13	0.38	2.9	0.052	0.045	0.86
GI	0.028	0.24	8.6	0.011	0.028	2.6
IC/PP1	0.048	0.11	2.2	0.019	0.012	0.65
IF	0.035	0.13	3.6	0.014	0.015	1.1
ILFB	0.033	0.34	10	0.013	0.040	3.1
ISTR	0.023	0.059	2.6	0.0089	0.0069	0.78
IT1	0.012	0.29	24	0.0047	0.034	7.2
IT2	0.098	0.40	4.1	0.039	0.047	1.2
IT3	0.025	0.55	22	0.010	0.064	6.4
PIFE1	0.017	0.25	15	0.0065	0.030	4.6
PIFE2	0.013	0.34	26	0.0051	0.040	7.7
PIFE3	0.035	0.045	1.3	0.014	0.0053	0.38
PIFI1	0.065	0.12	1.8	0.026	0.014	0.53
PIFI2	0.19	0.31	1.6	0.076	0.036	0.47
PIT	0.047	0.14	2.9	0.019	0.016	0.85
PP/PP2	0.0085	0.057	6.7	0.0033	0.0066	2.0
TA	0.035	0.17	4.9	0.014	0.020	1.4
**Total**	**2.7**	**8.6**		**1.0**	**0.98**	
		**Mean**	**3.2**			

*Note*: MTU masses are compared as percentages of body mass (M_b_) and as percentages of total MTU mass. See Table 4 caption for further details.

Our overall findings support our expectation (see Introduction) that *Postosuchus* had larger hindlimb muscles than in a similar‐sized crocodile, by a factor of more than two. This difference surely arises from the former's larger pelvic appendages but also might relate to bipedalism. Interestingly, the CFL was not exceptionally large in our *Postosuchus* model: only 1.7 times larger versus body mass, and only 0.51 times the size relative to total muscle mass; surely caused by the relatively smaller tail in *Postosuchus* (Table 3; less than half the relative mass of a representative crocodile's tail). Notably, these differences in muscle mass proportions do not all (except for PIFE1 and PIFE2; clearly resulting from the long pubes in *Postosuchus*) correspond closely to the MTU length differences described above; especially the longer CFL MTU length in *Postosuchus*. Hence, the CFL was relatively long but slender in the latter. If *Postosuchus* was bipedal, our findings for the CFL's size are somewhat problematic for the hypothesis that archosaurian bipedalism intrinsically involved an enlarged CFL (Persons & Currie, 2017).

## 4 CONCLUSIONS

Here, we developed and analyzed a 3D musculoskeletal model to aid in a critical review of whether *Postosuchus* was (1) quadrupedal or bipedal and (2) plantigrade or digitigrade. These two main questions involve exploring how musculoskeletal morphology correlates with those functions, including the usage of separate 3D models of *Postosuchus* with different plantigrade versus digitigrade bone articulations and comparison of their resulting ROMs. In the reconstruction of our musculoskeletal model, we found the pelvic limb myology of *Postosuchus* to have a mix of traits that are plesiomorphic archosaurian (e.g., AMB, ADD2 and IF insertions), derived suchian (e.g., ‘lumbar’ PIFI2 origin; concentrated ILFB insertion) more derived “rauisuchian” (e.g., supra‐acetabular buttress related to an expanded IT 2 origin) and singularly derived features (e.g., lateralized CFB and PIFI1 origins; reduced digit V leading to concentration of GE, GI, and FL insertions onto the calcaneal tuber; expanded FHL origins); but a lack of obvious synapomorphies with “sphenosuchians.” Additionally, our functional analysis found limited hip abduction caused by the supra‐acetabular crest, and other traits in the distal hindlimb that favored more parasagittal joint motions.

We found that it is far from conclusive that *Postosuchus* was habitually bipedal or digitigrade. Importantly, the model's COM is positioned considerably cranial to the hips, so bipedalism might have been difficult to achieve. The body segment dimensions are also more characteristic of a quadruped. But the humerus and femur circumferences instead support the inference of bipedalism. Our morphofunctional analyses of potential digitigrade versus plantigrade poses are somewhat ambiguous but the more plesiomorphically robust pes of *Postosuchus* is consistent with plantigrady. We reviewed evidence proposed for digitigrady (see Gauthier et al., 2011; Schachner et al., 2020) in taxa such as *Postosuchus* and found that inconclusive. Our analyses of muscle MMAs found trade‐offs between plantigrady versus digitigrady. Some of the above evidence must be introducing a misleading signal, although facultative bipedalism might explain the discordance. Furthermore, the functional morphology of the manus is more characteristic of quadrupeds despite the small size of the manus.

To answer our third question about pelvic limb muscle specializations in *Postosuchus*, we estimated pelvic limb muscle sizes using a volumetric model based on our musculoskeletal model (estimated 396.2 kg) and compared the results with dissection‐based muscle architecture data from an extant adult crocodile of similar body mass (278 kg). We confirmed our predictions that *Postosuchus* had relatively larger pelvic limb muscles, finding that they were not only more massive but also longer, corresponding to the relatively longer hindlimbs and more expansive pelvis of *Postosuchus*. The larger pubes and resulting great masses and lengths of the PIFE1 and PIFE2 muscles stand out as remarkable specializations that (based on typical archosaurian MMAs for these muscles; e.g., Hutchinson et al., 2005; Bates et al., 2015; Allen et al., 2021; Cuff et al., 2022) would have given the capacity for considerable hip flexor, adductor, and external rotator moment generation from these two muscles. The long but relatively low‐mass CFL (related to the gracile tail; and a more cranially positioned COM) is good cause to search further across potentially bipedal (and quadrupedal outgroup) archosaurs to test if a large CFL directly relates to bipedalism (Persons & Currie, 2017).

Solving whether *Postosuchus* was quadrupedal or bipedal, plantigrade or digitigrade, needs ichnological, simulation, or robotics evidence; or perhaps all of these (e.g., Nyakatura et al., 2019). Our biomechanical musculoskeletal model forms a useful foundation for such deeper analyses of locomotor function in *Postosuchus*. Furthermore, the model we provide could easily be revised in light of new findings or different interpretations of musculoskeletal form and function. Improved insights into the locomotion of *Postosuchus* could have broader application beyond understanding how this unusual large Triassic carnivore functioned in its environment. In particular, the controversies about derived traits of bipedalism and digitigrady apply to other “rauisuchians” with less extreme limb morphologies. Taxa such as *Batrachotomus* and *Prestosuchus* have sometimes been suggested as being at least facultatively bipedal (Grinham et al., 2019; Liparini, 2011) and/or digitigrade. If *Postosuchus* was bipedal and/or digitigrade, then that could strengthen inferences of those stances in other “rauisuchians,” and ideally would lead to identification of morphofunctional traits that are causative of those stances, and thereby would have useful application to other taxa and to major questions about locomotor evolution (e.g., Allen et al., 2021; Bates et al., 2015; Bates & Schachner, 2012; Charig, 1972; Cuff et al., 2022; Demuth et al., 2023; Gauthier et al., 2011; Grinham et al., 2019; Hutchinson, 2006; Kubo & Kubo, 2012; Nesbitt & Norell, 2006; Parrish, 1987; Persons & Currie, 2017; Sullivan, 2015).

## AUTHOR CONTRIBUTIONS

Conceived and designed study: JRH; Funding: JRH; Digitizing bones: RP, FC, MH, and EF; Model construction: JRH, EF, MH, TD, and OED; Data analysis: JRH; Paper writing: JRH; Paper editing: all authors.

## Supporting information


**Figure S1.** Left forelimb joint coordinate systems (JCSs) for the *Postosuchus* model, in oblique craniolateral view. Joints (shoulder, elbow, wrist, and MCP3 = third metacarpophalangeal) are labelled next to their flexion/extension axes. Red, green, and blue colored axes (*x*, *y*, *z*, respectively) are long‐axis rotation, adduction/abduction, and flexion/extension as labelled (following Gatesy et al., 2022). The limb is in the reference pose (all angles = 0°). Arrows point toward positive values of angles.
**Figure S2**. *Poposaurus gracilis* specimen YPM 57100 (panels A–D; digitigrade pose) and *Postosuchus* composite model (panels E–H; plantigrade pose) for comparison and contrast; left pes in various views. A and E, dorsal; B and F, ventral; C and G, lateral; D and H, medial. Scale bars are 10 cm for each specimen.
**Figure S3**. *Postosuchus* plantigrade left and right crus and pes regions in cranial view, showing “toed‐out” orientation. Not to scale.
**Figure S4**. Simple estimates of left shoulder joint ROMs for *Postosuchus*; and related morphological traits. Minimal and maximal angles for: A, shoulder flexion (−50°; lateral view; extension is 0°); B, shoulder extension (80°; lateral view); C, shoulder abduction (50°; caudal view); D, shoulder internal LAR (−40°; craniolateral view); and E, shoulder external LAR (30°; dorsal view). Red arrows indicate articular interactions (contact/disarticulation) used to approximate ROM limits. Not to scale.
**Figure S5**. Simple estimates of left lower forelimb joint ROMs for *Postosuchus*; and related morphological traits. Minimal and maximal angles for: A, elbow flexion (−120° in lateral view; extension is 0°); B, wrist dorsiflexion (−65°; lateral view); C, wrist palmarflexion (90°; lateral view); D, third metacarpophalangeal joint flexion (palmarflexion −90°; craniolateral view); and E, third metacarpophalangeal joint extension (dorsiflexion −110°; caudomedial view). Red arrows indicate articular interactions (contact/disarticulation) used to approximate ROM limits. As with the pes, our model solely used the third metacarpophalangeal joint, and because scan resolution was not ideal to separate joint surfaces and the metacarpals are not all the same lengths and orientations, digits I, II, IV, and V may rotate in unrealistic ways versus digit III. Not to scale.

## Data Availability

Photogrammetric data for the postcranial elements are available at MorphoSource.org (femora: https://www.morphosource.org/projects/000407464; other files: https://www.morphosource.org/projects/000853404). The OpenSim and volumetric muscle Maya musculoskeletal models and meshes are at Figshare (doi: 10.6084/m9.figshare.31228246; https://figshare.com/s/44929fa320ca0588b9c2). All other files are in the Supporting Information.
