# Supplementary material for: Functional morphology and biomechanics of the locomotor apparatus in the large Late Triassic carnivore Postosuchus kirkpatricki (Archosauria: Rauisuchidae)
Source: J Anat. 2026 Jun 5:10.1111/joa.70189. Online ahead of print. doi: 10.1111/joa.70189 (PMC13398517; doi:10.1111/joa.70189)
Supplement: Supplementary file 1 — Figure S1. Left forelimb joint coordinate systems (JCSs) for the Postosuchus model, in oblique craniolateral view. Joints (shoulder, elbow, wrist, and MCP3 = third metacarpophalangeal) are labelled next to their flexion/extension axes. Red, green, and blue colored axes (x, y, z, respectively) are long‐axis rotation, adduction/abduction, and flexion/extension as labelled (following Gatesy et al., 2022). The limb is in the reference pose (all angles = 0°). Arrows point toward positive values of angles. Figure S2. Poposaurus gracilis specimen YPM 57100 (panels A–D; digitigrade pose) and Postosuchus composite model (panels E–H; plantigrade pose) for comparison and contrast; left pes in various views. A and E, dorsal; B and F, ventral; C and G, lateral; D and H, medial. Scale bars are 10 cm for each specimen. Figure S3. Postosuchus plantigrade left and right crus and pes regions in cranial view, showing “toed‐out” orientation. Not to scale. Figure S4. Simple estimates of left shoulder joint ROMs for Postosuchus; and related morphological traits. Minimal and maximal angles for: A, shoulder flexion (−50°; lateral view; extension is 0°); B, shoulder extension (80°; lateral view); C, shoulder abduction (50°; caudal view); D, shoulder internal LAR (−40°; craniolateral view); and E, shoulder external LAR (30°; dorsal view). Red arrows indicate articular interactions (contact/disarticulation) used to approximate ROM limits. Not to scale. Figure S5. Simple estimates of left lower forelimb joint ROMs for Postosuchus; and related morphological traits. Minimal and maximal angles for: A, elbow flexion (−120° in lateral view; extension is 0°); B, wrist dorsiflexion (−65°; lateral view); C, wrist palmarflexion (90°; lateral view); D, third metacarpophalangeal joint flexion (palmarflexion −90°; craniolateral view); and E, third metacarpophalangeal joint extension (dorsiflexion −110°; caudomedial view). Red arrows indicate articular interactions (contact/disarticulation) used to approxi [file JOA-9999-0-s001.zip › joa70189-sup-0004-FigureS1-S5.docx]

**Supplementary Information for**

Hutchinson, J.R., Faughey, E., Humpage, M., Dupuis, T., Demuth, O.E., Pintore, R., Clarac, F. 2026. Functional morphology and biomechanics of the locomotor apparatus in the large Late Triassic carnivore *Postosuchus kirkpatricki* (Archosauria: Rauisuchidae). *Journal of Anatomy* manuscript 5401296.

**Contains Supplementary Figures S1-S5**


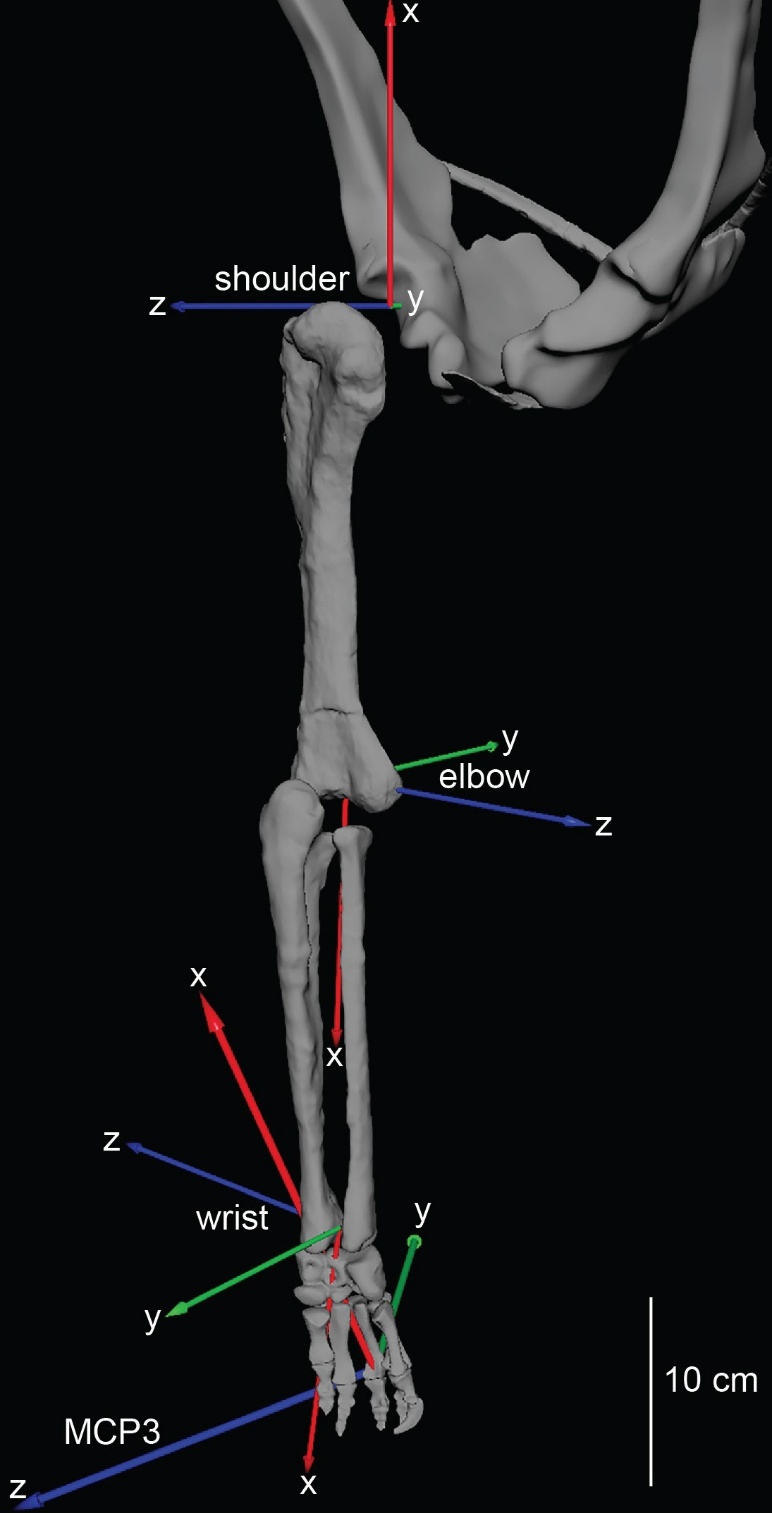


**Figure S1.** Left forelimb joint coordinate systems (JCSs) for the *Postosuchus* model, in oblique craniolateral view. Joints (shoulder, elbow, wrist and MCP3 = third metacarpophalangeal) are labelled next to their flexion/extension axes. Red, green and blue coloured axes (x, y, z respectively) are long-axis rotation, adduction/abduction and flexion/extension as labelled (following Gatesy et al., 2022). The limb is in the reference pose (all angles = 0°). Arrows point toward positive values of angles.


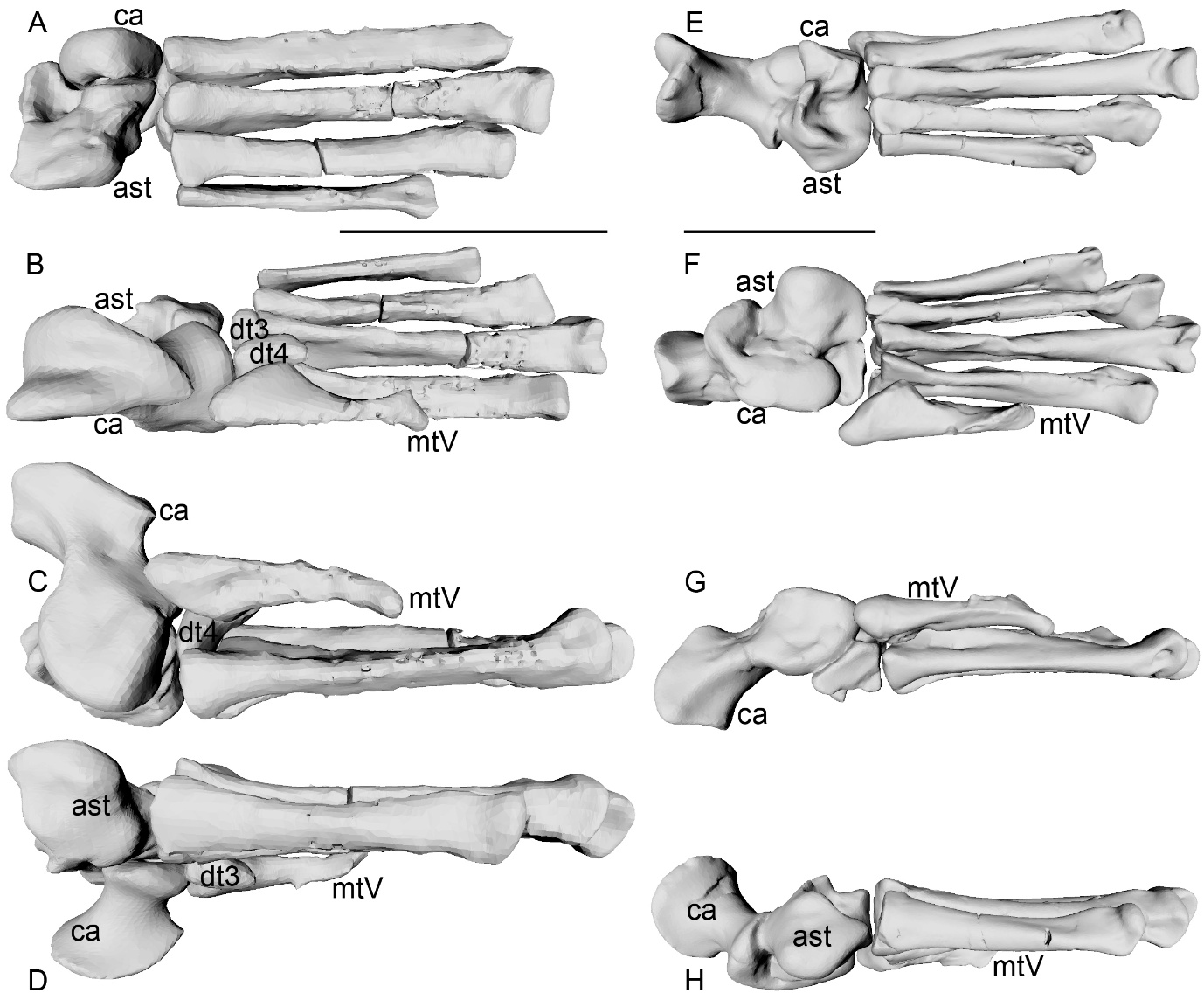


**Figure S2.** *Poposaurus gracilis* specimen YPM 57100 (panels A-D; digitigrade pose) and *Postosuchus* composite model (panels E-H; plantigrade pose) for comparison and contrast; left pes in various views. A and E, dorsal; B and F, ventral; C and G, lateral; D and H, medial. Scale bars are 10cm for each specimen.

**
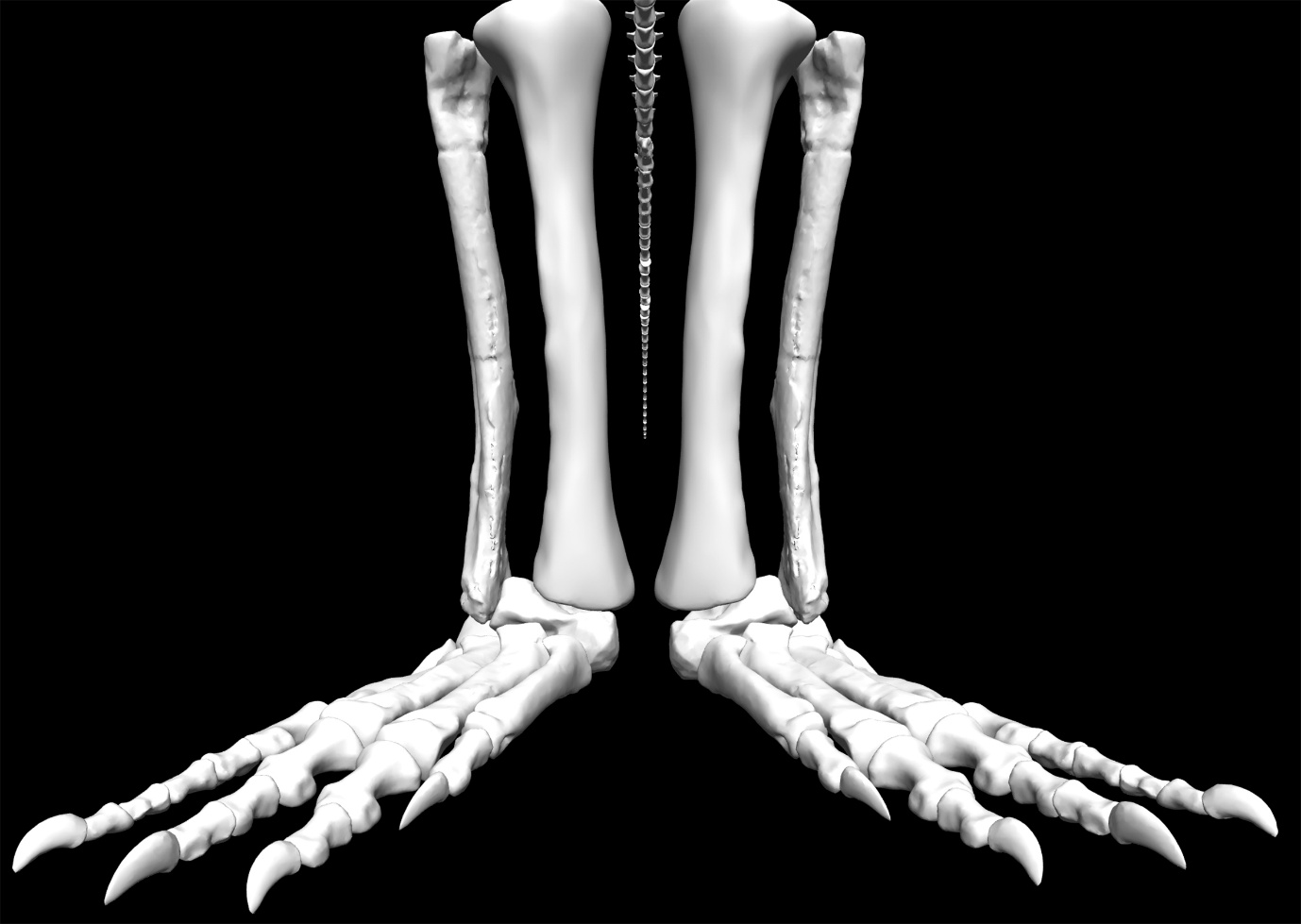
**

**Figure S3.** *Postosuchus* plantigrade left and right crus and pes regions in cranial view, showing ‘toed-out’ orientation. Not to scale.

**
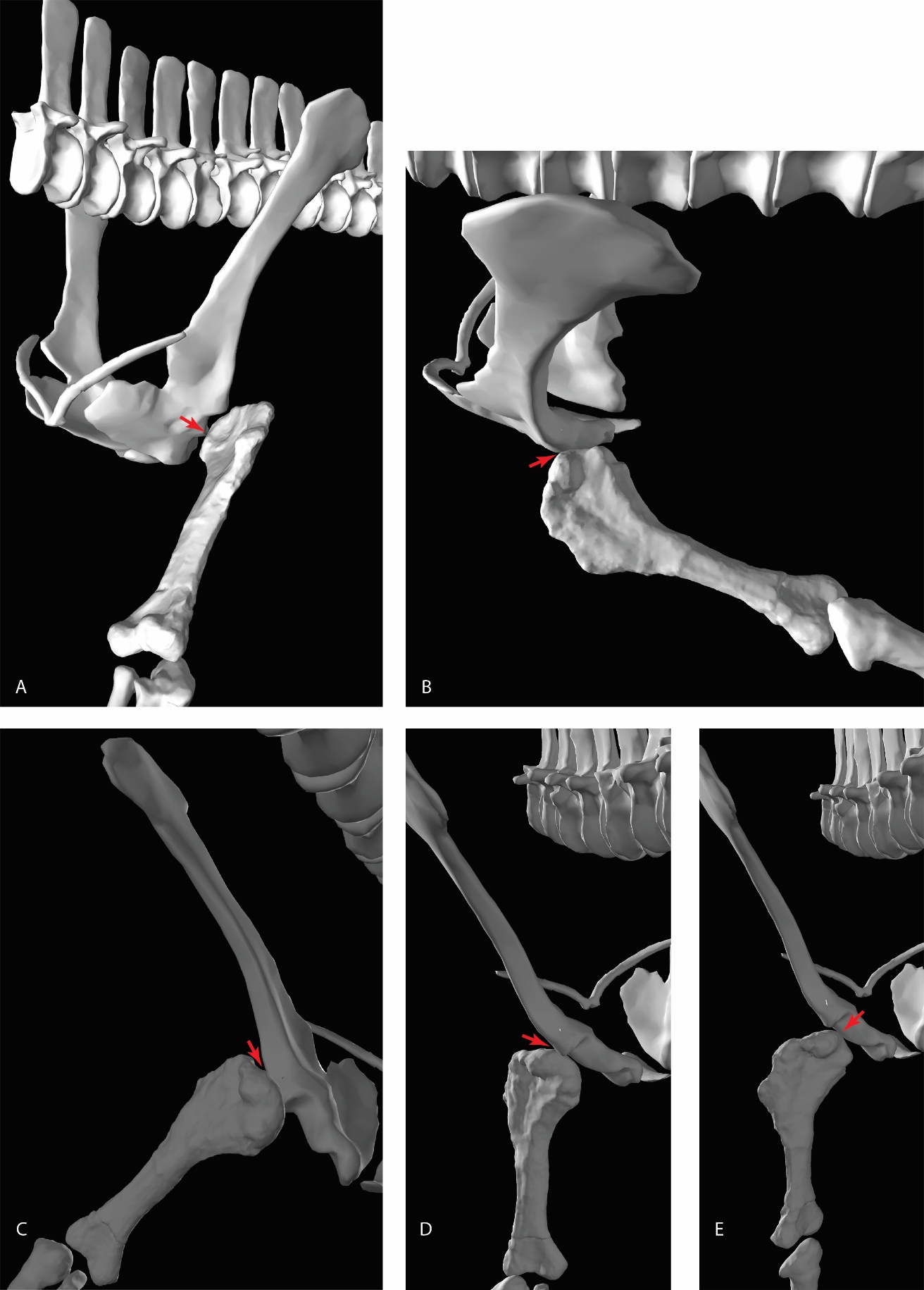
**

**Figure S4.** Simple estimates of left shoulder joint ROMs for *Postosuchus*; and related morphological traits. Minimal and maximal angles for: A, shoulder flexion (-50°; lateral view; extension is 0°); B, shoulder extension (80°; lateral view); C, shoulder abduction (50°; caudal view); D, shoulder internal LAR (-40°; craniolateral view); E, shoulder external LAR (30°; dorsal view). Red arrows indicate articular interactions (contact/disarticulation) used to approximate ROM limits. Not to scale.

**
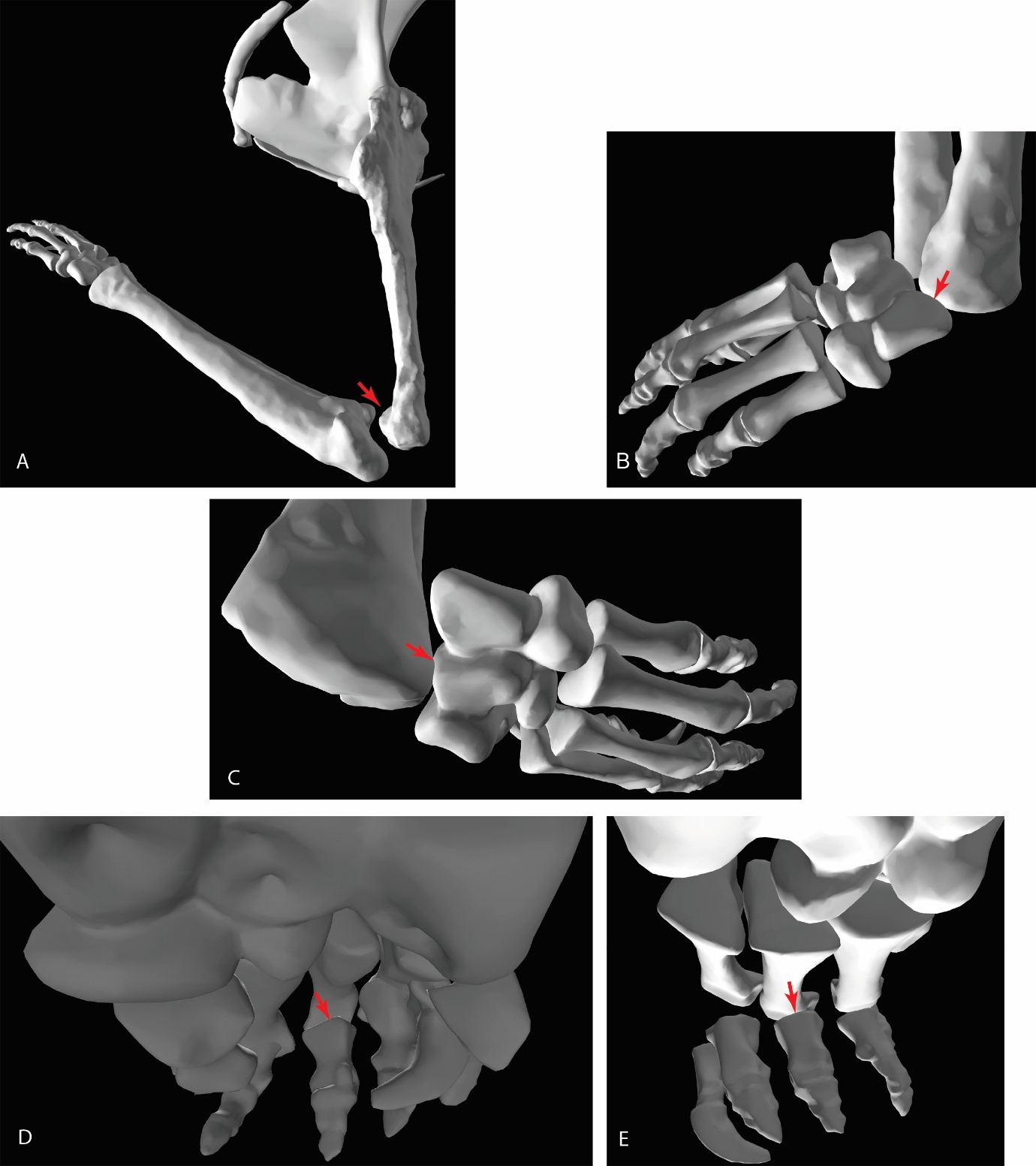
**

**Figure S5.** Simple estimates of left lower forelimb joint ROMs for *Postosuchus*; and related morphological traits. Minimal and maximal angles for: A, elbow flexion (-120° in lateral view; extension is 0°); B, wrist dorsiflexion (-65°; lateral view); C, wrist palmarflexion (90°; lateral view); D, third metacarpophalangeal joint flexion (palmarflexion -90°; craniolateral view); E, third metacarpophalangeal joint extension (dorsiflexion -110°; caudomedial view). Red arrows indicate articular interactions (contact/disarticulation) used to approximate ROM limits. As with the pes, our model solely used the third metacarpophalangeal joint, and because scan resolution was not ideal to separate joint surfaces and the metacarpals are not all the same lengths and orientations, digits I, II, IV and V may rotate in unrealistic ways vs. digit III. Not to scale.
